# Implications of immersive technologies in healthcare sector and its built environment

**DOI:** 10.3389/fmedt.2023.1184925

**Published:** 2023-09-20

**Authors:** Eunsil Yang

**Affiliations:** Healthcare Facilities, Bartlett School of Sustainable Construction, University College London, London, United Kingdom

**Keywords:** immersive technology, extended reality (XR), medical extended reality (MXR), virtual reality (VR), digital health, health metaverse, therapeutic environment, future healthcare ecosystems

## Abstract

**Objectives:**

This research focuses on how built environment experts can contribute to the MXR-enabled digital innovation as part of the multidisciplinary team effort to ensure post-pandemic resilience in healthcare built environment. The goal of this research is to help healthcare providers, built environment experts, and policy makers respectively: (1) Advocate the benefits of MXR for innovating health and social care; (2) Spark debate across networks of expertise to create health-promoting environment; and (3) Understand the overriding priorities in making effective pathways to the implementation of MXR.

**Methods:**

To highlight the novelty of this research, the study relies on two qualitative methodologies: exploratory literature review and semi-structured interviews. Based on the evaluation of prior works and cross-national case studies, hypotheses are formulated from three arenas: (1) Cross-sectional Initiatives for Post-pandemic Resilience; (2) Interoperability and Usability of Next-gen Medicines; and (3) Metaverse and New Forms of Value in Future Healthcare Ecosystems. To verify those hypotheses, empirical findings are derived from in-depth interviews with nine key informants.

**Results:**

The main findings are summarized under the following three themes: (1) Synergism between Architecture and Technology; (2) Patient Empowerment and Staff Support; and (3) Scalable Health and Wellbeing in Non-hospital and Therapeutic Settings. Firstly, both built environment and healthcare sectors can benefit from the various capabilities of MXR through cross-sectional initiatives, evidence-based practices, and participatory approaches. Secondly, a confluence of knowledge and methods of HCI and HBI can increase the interoperability and usability of MXR for the patient-centered and value-based healthcare models. Thirdly, the MXR-enabled technological regime will largely affect the new forms of value in healthcare premises by fostering more decentralized, preventive, and therapeutic characteristics in the future healthcare ecosystems.

**Conclusion:**

Whether it's virtual or physical, our healthcare systems have placed great emphasis on the rigor of evidence-based approach linking health outcome to a clinical environment. Henceforth, built environment experts should seek closer ties with the MXR ecosystems for the co-production of scalable health and wellbeing in non-hospital and therapeutic settings. Ultimately, this is to improve resource efficiency in the healthcare sector while considering the transition of health resources towards *in silico* status by increasing the implementation of MXR.

## 1. Introduction

**Figure 1:**
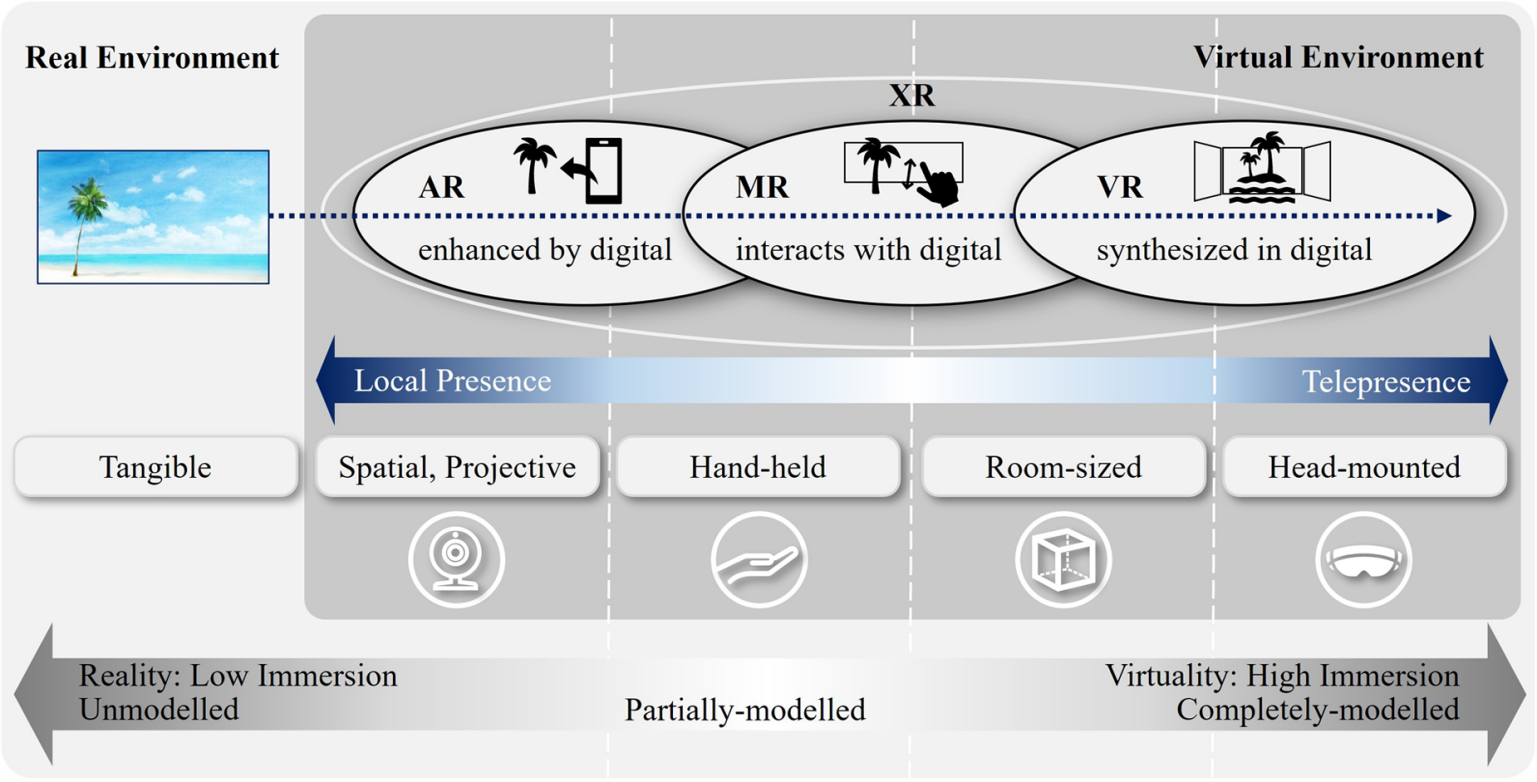
Reality-virtuality continuum of immersive technologies.

This research takes qualitative approaches to examine the Implications of Immersive Technologies in the Healthcare Sector and its Built Environment. By doing so, the research aims to shed light on how built environment experts can contribute to more resilient healthcare systems in the post-pandemic future. Harnessing digital technologies enables us to reimagine healthcare models and workforce agility (1–3) and remote care has become a major trend for public health services (4). In June 2022, the UK government issued the “Digital Revolution of Health and Social Care”, considering the rapid expansion of remote care to free up hospital space; save clinician time; and bust the COVID backlogs (5). Meanwhile, immersive technologies are becoming readily available for more interactive, engaging, and efficient medical practices. Figure 1 describes the properties of immersive technologies by juxtaposing two opposite ends—reality and virtuality (6, 7). In this research, immersive technologies will be used interchangeably with Extended Reality (XR), an umbrella term encapsulating Augmented Reality (AR), Virtual Reality (VR), and Mixed Reality (MR). Specifically, Medical Extended Reality (MXR) refers to XR in healthcare.

**Figure 2:**
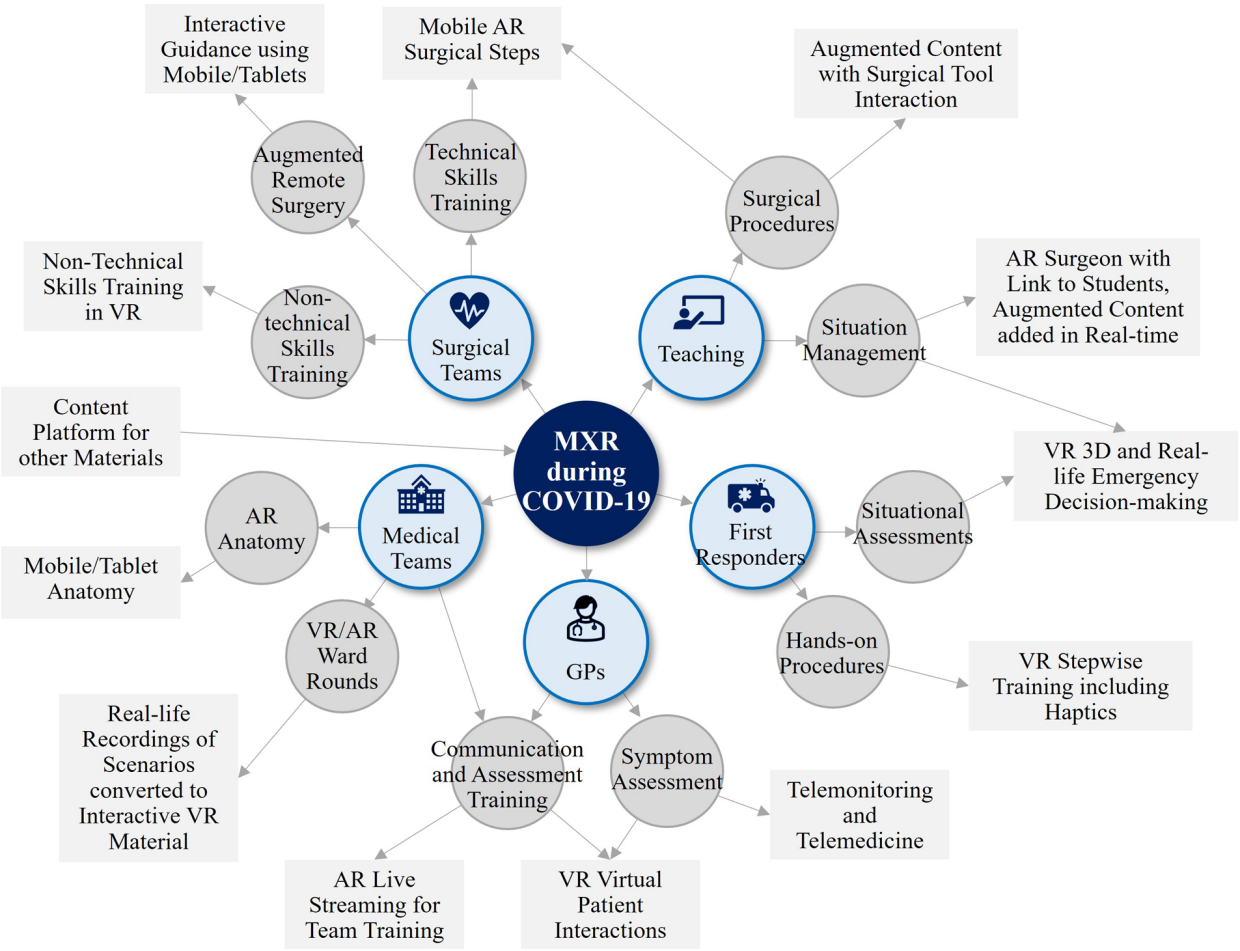
MXR during COVID-19 (14), showing suggested XR technologies and apps for a small portion of healthcare roles. Reproduced with permission of the copyright owner.

When considering the technology adoption rates of US households, it is expected that at least 70 million users will adopt XR within 6 years (8). Goldman Sachs Global Investment estimated the 2025 global XR market size by different use cases where the healthcare industry represents $5.1bn (9). Bloomberg UK forecasts that the global MXR market revenue growth will reach $14.06bn by 2030 due to the rising demand of accurate robotic surgery (10). Furthermore, the COVID-19 pandemic has propelled disruptive innovation to come up with new ways of delivering healthcare services. As climate change will cause more frequent viral outbreaks, XR will find a wider application in healthcare industry (11–13). In the UK, various XR technologies assisted healthcare roles during the COVID-19 epidemic (14) (see Figure 2). Also, an early adoption of XR addressed some of the biggest public health challenges from the COVID-19 pandemic, according to Health Education England (15, 16).

MXR is one of the key digital health technologies that can reinforce the sociological characteristics of future healthcare systems. For instance, National Health Services (NHS) in the UK operates Health Call and Virtual Wards to increase access to digital healthcare solutions, thus mitigating health inequities across the country. Babylon, a digital health company, launched the AI-powered triage tool in Rwanda to improve the country's healthcare systems by digitizing them (17). Particularly, MXR can help fight a chronic healthcare issue of staff retention. Broomx Technologies, the Spanish immersive solutions company, supports the mental health and wellbeing of healthcare workers through their multi-sensory projects, called “Humanization of Hospital Spaces” (18). In our rapidly ageing society, many experts and researchers have predicted that the next generation of health and social care will be shifted to “From-Facility-To-Home” or “Hospital-at-Home” models, especially for older adults. Moreover, XR can benefit the elderly and their caregivers by increasing their autonomy in lives, thus reducing social and economic burden of the entire society (19–25).

**Figure 3:**
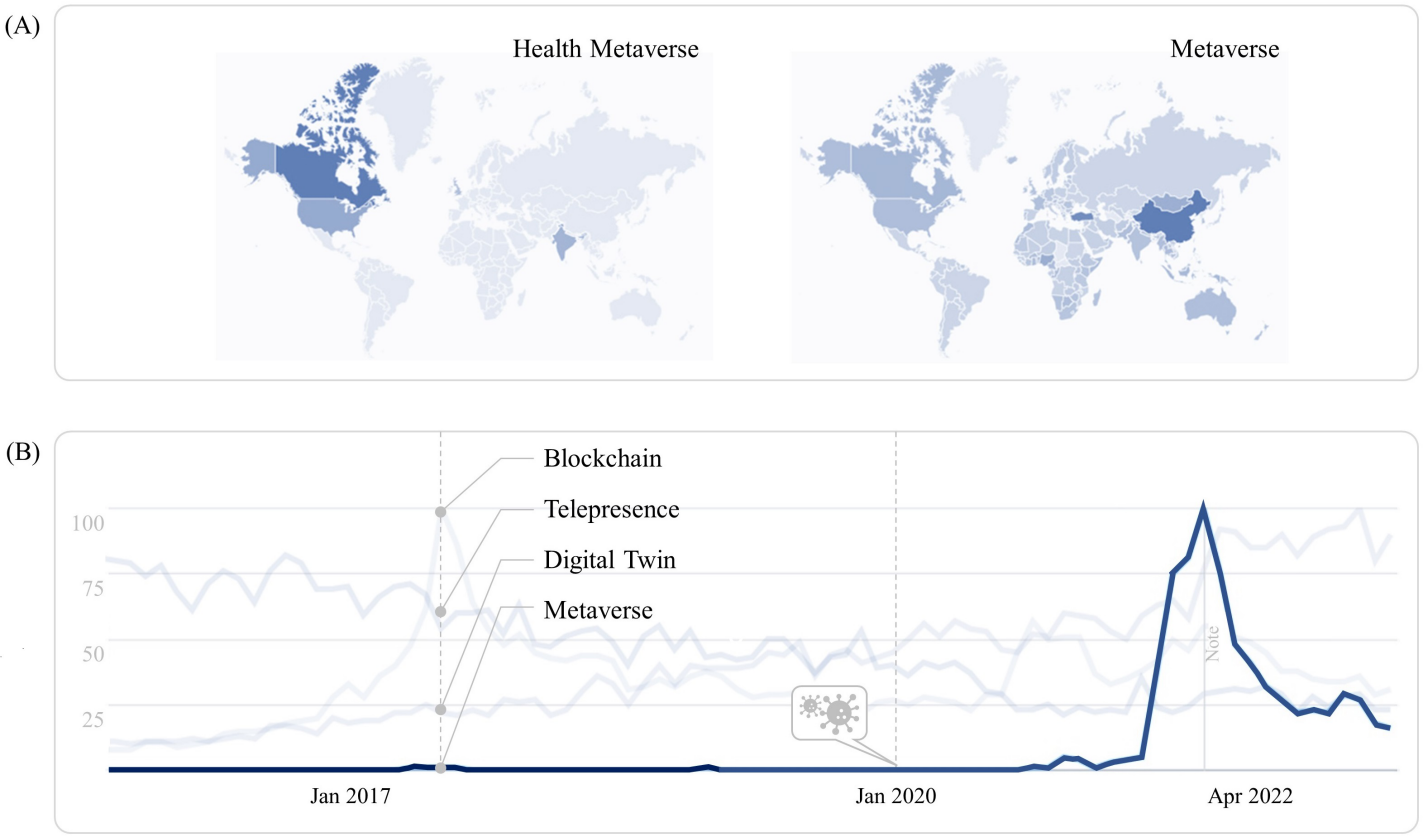
Google trends of health metaverse in 2015–2022 (30), showing (**A**) the regional share of interest in health metaverse and metaverse; (**B**) the interest over time in blockchain, telepresence, digital twin, and metaverse. Reproduced with permission of the copyright owner.

Coupled with the exponential growth of MXR, the healthcare metaverse will soon provide an open platform for real-time collaboration (26–29). Although it is still in its embryonic stage, the metaverse as a nascent paradigm gains more credibility with the increasing prevalence of three major technologies: telepresence, digital twin, and blockchain (30–32) (see Figure 3). Consequently, healthcare facilities must evolve in response to this technological disruption because a new modality of healthcare will later transform its built environment when XR finally becomes integral to the future healthcare ecosystems. Yet, most of the current academic discourse and market research on MXR were conducted from the healthcare providers’ perspectives. With increasing healthcare spending by governments and real-world deployments of MXR, it is critical to identify an agenda for future research to consider the ripple effect of immersive technologies on healthcare services and facilities. The research aims to address this gap by detailing a coherent framework to identify the roles and responsibilities of built environment experts as part of the multidisciplinary team effort for more effective implementation of MXR. Accordingly, three critical research questions arise as to:

(i) How does the MXR contribute to the advancement of healthcare services and facilities to improve population health and wellbeing in the post-pandemic era? Can built environment experts benefit from the expansion of MXR?
(ii) What are the risks and limitations that hamper a widescale adoption of MXR in healthcare sector? Can built environment experts mitigate against some of those obstacles?
(iii) What are the roles and responsibilities of built environment experts in creating a long-standing value chain of healthcare premises undergoing transformation within the future healthcare ecosystems reformed by MXR?

To answer these, the research is organized into six main stages. Following the introduction, related work establishes three hypotheses through the theoretical frameworks that are identified from literature review. The qualitative methodology was taken to gather results from in-depth interviews with nine key informants. Then the discussion lays down the key findings which are summarized with the suggestions for future research in the conclusion. As a result, this research aims to help healthcare providers, built environment experts, and policy makers respectively: (1) Advocate the benefits of MXR for innovating health and social care; (2) Spark debate across networks of expertise to create health-promoting environment; and (3) Understand the overriding priorities in making effective pathways to the implementation of MXR.

## 2. Related work

### 2.1. Objectives and search criteria

This section provides the current context of academic and industry perspectives on the MXR through an exploratory literature review of prior research and cross-national case studies. Many researchers have called for a transdisciplinary culture to maximize the potential of MXR (16, 33), however the merits of prior conceptualizations or methodologies to define the roles and responsibilities of built environment experts in this nascent technological regime are still limited. Given that, this review strives to determine the extent to which any interpretable pattern elicits the interrelations between MXR and built environment and their impacts on the future healthcare ecosystems.

The review commenced with Google Search Engine, Google Scholar, and reputable academic databases, then completed with the last search on 15th August 2022. Since the most MXR research has been published in conference papers (90.05%) rather than in journals (9.95%) (34), data was also collected from grey literature. The search terms included “XR”, “MXR”, and “Immersive Technologies” by limiting the results to the healthcare and AEC (Architecture, Engineering and Construction) industry. Only English language publications between 1990 and 2022 are considered to obtain globally recognized work while capturing the evolution of research topic. In light of research questions, the findings can be grouped into three arenas: (1) Cross-sectoral initiatives for Post-pandemic Resilience; (2) Interoperability and Usability of Next-gen Medicines; and (3) Metaverse and New Forms of Value in Future Healthcare Ecosystems.

### 2.2. Cross-sectoral initiatives for post-pandemic resilience

To avoid conceptual confusion and unclear demarcations, the latest classification of XR-related technologies reviews their typical usage contexts. The distinction between AR and VR is made based on whether the physical environment is part of user experiences or not (7). Namely, AR with local presence can be implemented everywhere, whereas VR with telepresence is to be operated with Head-mounted Displays (HMDs) in specific settings. Roughly speaking, MXR has three different modes of operation: in-bed, in-lab, and surgical session (35–37). Although the most prevalent modality of XR is still a mix of physical and virtual components, given the fact that *in vivo* exposure is yet inconvenient, expensive and time consuming, MXR will continuously replace and repeat the *in vivo* experience with forms of *in silico* contact (38, 39).

Many recent studies have focused on the roles of XR in enhancing health and social care (15, 40, 41). But at the same time, XR continues to find a home in the built environment sector for healthcare facilities development (42–44). MXR's diverse opportunities can be categorized into eight domains: (a) Enhancing surgical procedure and pain management; (b) Advancing design, construction and management of healthcare facilities; (c) Training and educating healthcare professional and patient; (d) Diagnosing and treating disorder and pathology; (e) Assisting physical impairment and rehabilitation; (f) Promoting mental health and wellbeing; (g) Ensuring healthy living for the elderly; and (h) Analyzing health data (40, 42, 45–52). Table 1 comprises MXR case studies based on their contemporaneity, practicality, and implications to the healthcare built environment.

**Table 1:** MXR opportunities.

Domain	Project details	Opportunities	Implications to built environment
(a)	VR-Nerve Block, Northumbria NHS Trust, UK (40)	Fewer opiate medications during surgery	Create virtual space to distract patients
(b)	VR Walkthrough, Stantec, US (42)	360-degree tour helps people visualize clinical spaces	Reshaping healthcare design practice with better decision-making
(c)	CTCA Scan Training, GE & Immerse, UK & US (45)	Simulation reduces onsite training, costs, and risks to patients	Substitute physical environment with 24/7 online access
(d)	gameChange, NIHR, UK (46)	Cognitive-behavioral therapy for patients with psychosis	User-centered design of imaginal exposure therapy
(e)	Canetroller, Microsoft, US (47)	Enabling people with visual impairment to navigate through outdoor/indoor scenes	Unhindered area for XR trials
(f)	Recharge rooms, Mount Sinai, US (48)	Multi-sensory experience to support health workers	Transform underused hospital space into regenerative purposes
(g)	LookBack, Virtue Health, UK (49)	Reminiscence therapy for dementia sufferers	Physical objects and space can enhance experience
(h)	VROOM, Western Sydney University, Australia (50)	Oncology data model for patient’s cohort	Unified environment with XR-related devices and technologies

A considerable number of MXR studies are published on medical training, education, and practice (14, 53–55). Especially, XR can effectively treat mental health problems and behavior disorders with a high degree of control over therapeutic experiences (38, 56–58). For example, Oxford Psychiatry has experimented with VR in helping people with psychosis. And an automated cognitive-behavioral session benefited patients with severe agoraphobic avoidance since it could be conducted in the participant's home (46). Moreover, MXR has been supporting the wellbeing of frontline healthcare staff not just as a short-term response to the pressure of working through backlogs, but as a long-term commitment to mitigating the global crisis of healthcare workforce (59–62).

The successful XR adoption must start with the building blocks of infrastructure and ecosystems (63). For this, several studies highlight the importance of transdisciplinary collaboration with various experts across industries (53, 64). Particularly, the adoption of Participatory Design (PD) has been advocated to foster an understanding of different needs between the stakeholders in developing MXR applications (65). There are many precedents of incorporating XR into the PD for designing healthcare facilities to improve the workflow amongst various construction project teams (66–70). However, the reverse rarely exists. Most MXR publications do not suggest the potential contribution of built environment experts in contrast to the heavy involvement of computer scientists and product designers to solve complex medical challenges. In general, the XR ecosystems do not sufficiently take into account feedback from the AEC industry (34).

Greenleaf, a neuroscientist at Stanford University's Virtual Human Interaction Lab, commented that many MXR consultants are from the tech industry such as gaming or IT applications. The problem is that they do not quite understand what is truly needed for the XR ecosystems in relation to the clinical environment (39). Therefore, having a dialogue across multiple disciplines will help them understand how technology can fit into the healthcare ecosystems without becoming a barrier. Based on these observations, the first hypothesis as a conjectural statement to explain the first research question is formulated as follows.

**Hypothesis 1:** MXR requires cross-sectoral initiatives to become an integral part of healthcare systems for post-pandemic resilience. Built environment experts can improve clinical environment to ensure the best use of MXR. In tandem, the development of healthcare facilities can benefit from evidence-based practices and participatory approaches using the various capabilities of MXR.

### 2.3. Interoperability and usability of next-gen medicines

Despite numerous benefits afforded by MXR, its development as an interoperable medicine faces several challenges. Medical interoperability refers to timely, secure, and seamless digital flow for integrating electronic health data across different settings and systems for better patient experience and clinical outcome (71–73). It is anticipated that the horizontal convergence of interoperability and portability for XR will be achieved by 2030 to ensure its wide-scale deployment in healthcare (74, 75). Yet, optimizing XR opportunities in parallel to the healthcare requirements on a global scale necessitates the coherent frameworks of standardization to increase clinical interoperability in currently unregulated and fragmented XR industry (76). Specifically, the importance of the Centers for Excellence has been highlighted to exchange knowledge and methods for XR solutions between healthcare providers, tech industries, and academic researchers. This potential collaboration is concerned with addressing the most pressing issues such as global digital exemplars, product development, quality assurance, procurement frameworks, and distribution through virtual pharmacies (16, 39, 72, 73, 75, 76). By considering all these, Table 2 analyzes the risks and limitations when determining how best to incorporate XR to the healthcare sector.

**Table 2:** MXR challenges.

Types	Issues and mitigations
Technical evaluation (16, 39, 75, 76)	• Lack of device usability measurement and procedures
	→ Integrate XR properties (Image Quality, Human Factors, Tracking)
Clinical Validation (16, 39, 76)	• Lack of effective claims and safe premarket applications
	→ Control trials with clearly defined endpoints
Implementation Time (39, 72, 73, 75, 76)	• Delay in loading, troubleshooting, and licensing
	→ Better connectivity and standardization
Cost and Funding Mechanism (16, 75, 76)	• More affordable devices, but cost is still high
	→ Diversify funding options
Workforce (39, 75)	• Lack of skilled workforce to provide technical support
	→ Provide content update and ongoing learning
Resistance and Pushback (39)	• Resistance to adopt innovative technology
	→ Provide conclusive evidence of MXR efficacy
Governance (16, 75, 76)	• Duplicated effort without clear pathway mapping
	→ Standardization and Medical Devices Regulations
Digital Ethics (16, 75, 76)	• Cybersecurity, cyberprivacy and other consent issues
	→ Protection of personal health data (e.g., XR Safety Initiative, US)
Digital Inequalities (16, 75)	• Systematic digital exclusion of disadvantaged groups
	→ Improve device ownership, digital literacy and precarity

Further to that, how built environment experts can help to mitigate these issues will be examined in this stage by synthesizing research findings around the transdisciplinary effort of achieving better user experiences through Human-Computer Interaction (HCI). It is because that the success of MXR is highly dependent on the quality of Immersive Media Experience (IMEx) which is affected by Human Influential Factors (HIFs) such as “Immersion”, “Sense of Presence”, “Cybersickness”, as well as mental and emotional status on the user viewpoint (77). And identifying the use-related risks through psycho-physiological measures is vital not only to keep sustained growth but also to justify further investment on MXR. Specifically, MXR experiences should emulate the real-life interactions as much as possible through a superior user interface since “Cybersickness” is mainly caused by a discrepancy between the user's visual and vestibular sensory systems (53, 77–79).

Those XR interactions can be defined by three criteria: relationship, collaboration, and engagement. And, based on these criteria, it is identified that current XR interaction methods have six different types: tangible, collaborative, multimodal, device-based, sensor-based, and hybrid (80). Among these, multimodal interactions provided in the MXR applications are particularly important to enable patients’ physical and mental health needs to be closely interwoven through HCI. And the essence of HCI is in stimulating user's cognitive process with multi-sensory experience through the tri-dimensional representation of virtual environment, which consists of two key elements: system requirements and user concerns (78, 81, 82). In other words, multimodal interactions can significantly increase the usability of XR by the technologies of employing simultaneous human senses, but unfortunately those sensing technologies are still immature (83). Therefore, it is worth looking at other alternative measures to deal with such deficiency toward a more realistic representation of virtual environment.

VR for health-related research on how individuals perceive and respond to surrounding environment is inherently interdisciplinary (84). Spiegel, a leader of the world's largest virtual medicine labs in California, US, explained about the MXR's four therapeutic mechanisms: promoting cognitive flow; dampening inner pain signals; enhancing healthy body at tension; and strengthening self-identity. Especially, strengthening self-identity is about helping patients establish meaningful connections with the world around them (85). In this regard, it is noteworthy that such socio-spatial aspects of HCI have been rigorously investigated by architects for the design of individual buildings and urban spaces. Kirsh, a Canadian cognitive scientist, compared the different approaches of architects and HCI practitioners by defining three different interfaces: circular interactivity for classical HCI, network interactivity with context aware, and architectural interactivity based on social and embodied ecology. In his lecture titled “What Can HCI Learn from Architecture about Interaction?”, it was proposed that high-dimensional architectural conception can be a fertile ground for HCI (86).

**Figure 4:**
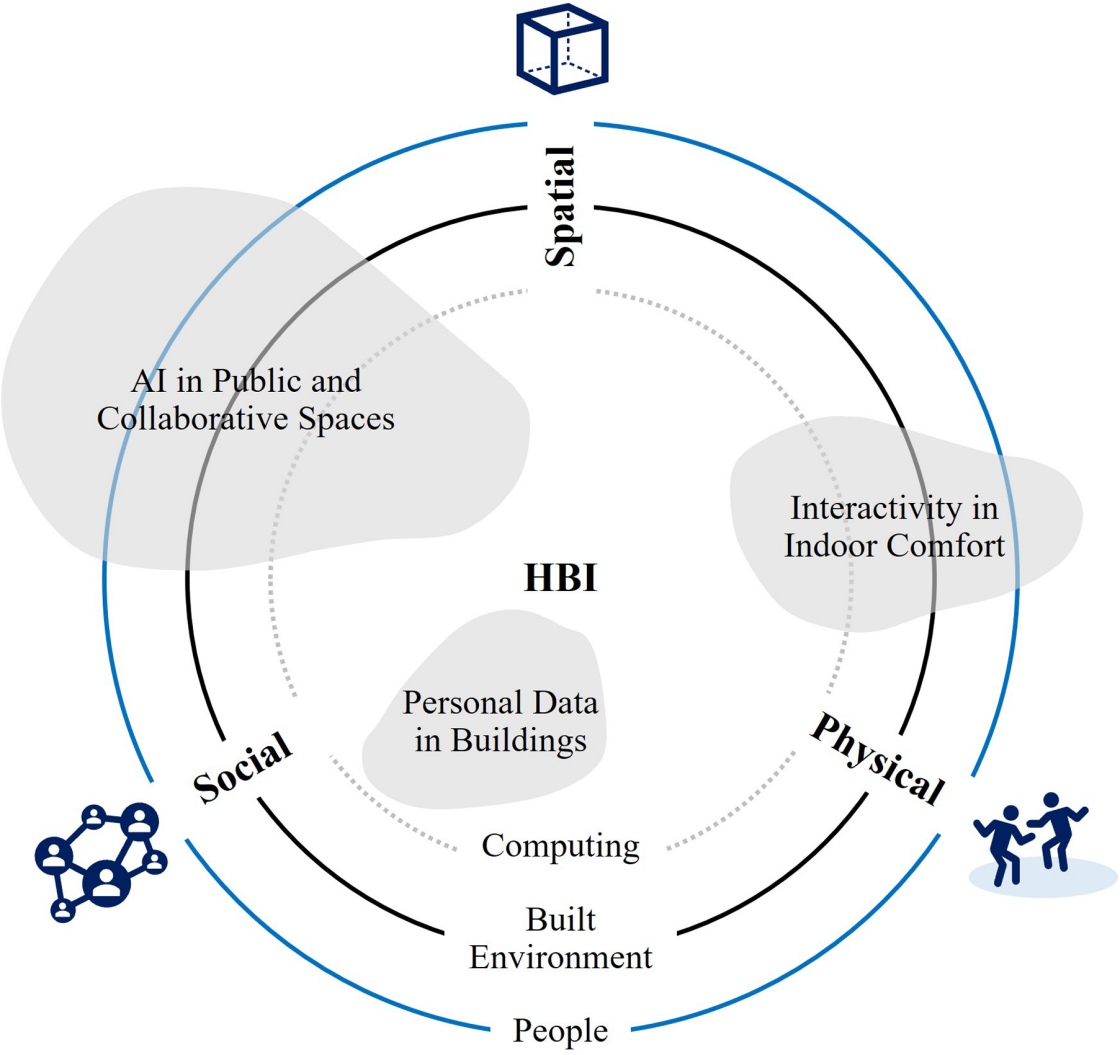
Three dimensions of HBI research (87), illustrating three concentric circles of “people”, “built environment”, and “computing” to describe how the HBI themes can operate between three dimensions: physical, spatial and social. Reproduced with permission of the copyright owner.

Similarly, the conception of Human-Building Interaction (HBI) has been introduced to address the complexity of human interactions with future built environment by implementing HCI principles into the domain of architecture and urban design (87) (see Figure 4). Also, the dynamics of whole-body interactions and social encounters in public spaces were studied by architects (88). The claims of these researchers are in line with the principles of Freedman's VR Exposure Therapy (VRET) that fosters a repertoire for coping with stressful social situations. In addition, how XR can affect human-scale spatial experiences and how digital and physical elements can be integrated in XR have been examined by built environment specialists (89, 90). In 2020, Arup proposed an incentivizing built environment that promotes active lifestyles with greater digital adoption as preventive approaches to wider health determinants. Here, the omnipresent layer of technologies in a ubiquitous built environment will empower people to take control of their own health and wellbeing (91). Based on these findings, the hypothesis derived from the second research question is as follows.

**Hypothesis 2**: Human-Computer Interaction (HCI), a foundation of the MXR, is rigorously studied by architects for optimal user experiences in the built environment. A confluence of knowledge and methods from two sectors can achieve more interoperable and usable MXR as the next-generation medicines to reinforce the patient-centered and value-based models of health and social care.

### 2.4. Metaverse and new forms of value in future healthcare ecosystems

Even before the COVID-19 pandemic, healthcare systems were already going through transformation due to the shocks and stresses induced by global issues such as climate change, demographic shifts, consumer expectations, and disruptive technologies (92–94). And the way we interact with the built environment has been changed by digital disruption, which led to the relocation and redesign of some forms of real estate (95). In the UK, most NHS properties face an unprecedented challenge to keep up with Net-Zero aspirations with an ambition to reach 80% reduction by 2040 (96, 97). Besides, Integrated Care Systems (ICSs) aim to combine health and care services through interoperable digital systems that require specialist estates capacity (98–100). Since 2013, the NHS Property Services has raised £418.5m through the disposal of 488 surplus properties because the strategic NHS estates planning is critical to generate not only a vital capital for the healthcare provision, but also an efficient value chain by reallocating socio-economic resources in a timely manner (101).

**Figure 5:**
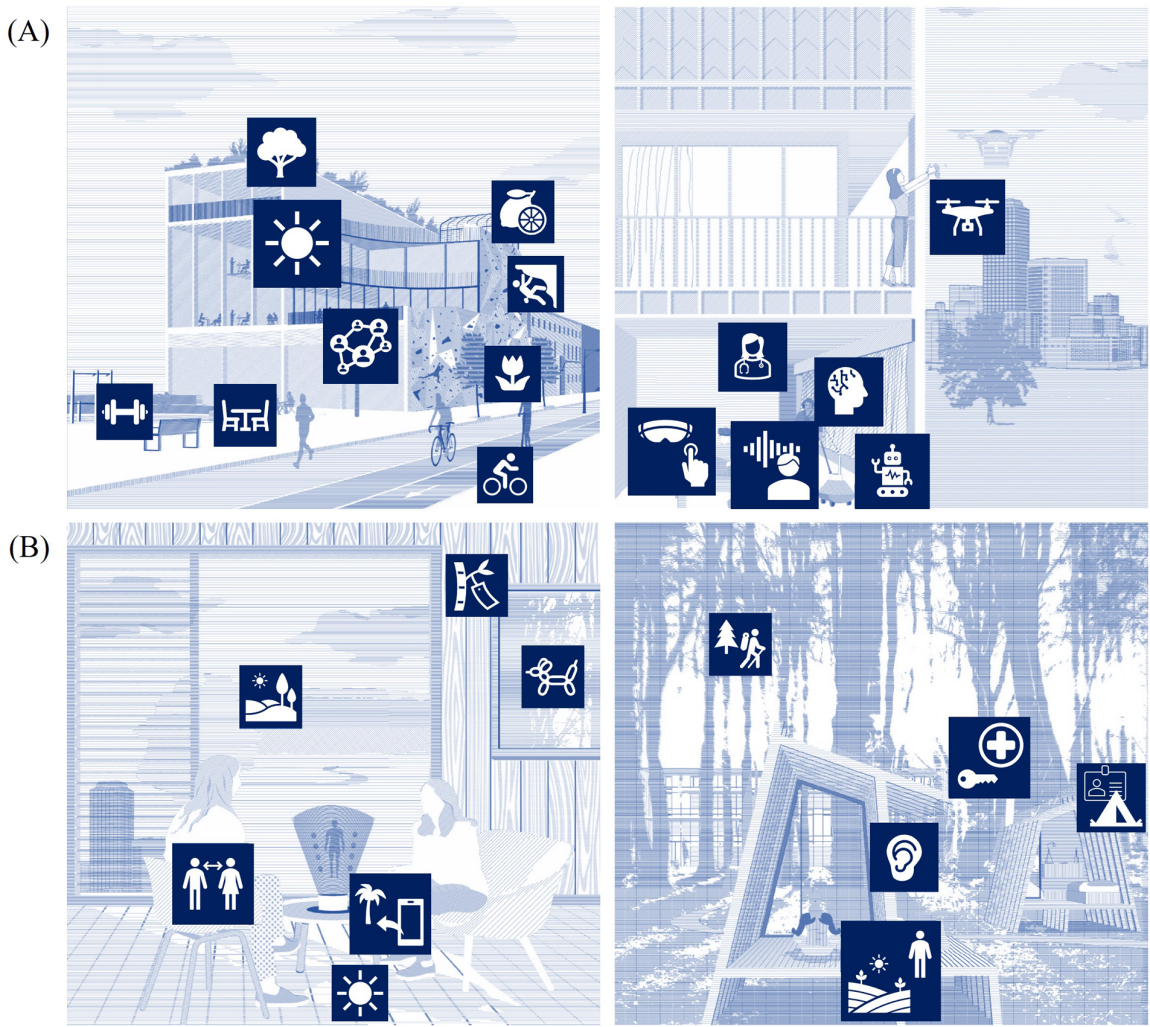
Blueprints of future healthcare ecosystems (91), rendering (**A**) incentivizing and ubiquitous infrastructure platforms; (**B**) humanized and personal healthcare built environment that are enhanced by digital technologies, including XR. Reproduced with permission of the copyright owner.

Henceforth, the NHS estates will be put further into a wider context in which healthcare premises will undergo significant alterations. In that sense, it is practically useful from a health economic perspective to envision how the healthcare facilities will evolve when MXR becomes an integral part of future healthcare ecosystems. As opposed to many digital technologies integrated to healthcare, built environment does not necessarily make them fit-for-purpose and thus not all buildings succeed in addressing two critical needs of ageing populations: personal autonomy and inclusivity. Therefore, to foster individual's health and wellbeing in the highly digitized healthcare systems, policies and interventions should entail a new design paradigm of “Therapeutic Architecture” to operate with people-centered and evidence-based discipline in harmony with psychology and physiology (102, 103). In the same vein, Arup envisaged that increased remote care will result in fewer visits to healthcare facilities. Instead, more therapeutic spaces will incorporate natural elements into the humanized and personal space (89) (see Figure 5).

Meanwhile, there has been a gradual transition towards more virtual-oriented methods to complement traditional healthcare systems. Digital Therapeutics (DTx), as a sub-category of the Digital Health landscape, will bring the healthcare systems into a digital future with emphasis on cost efficiency and changing lifestyles (104–106). DTx are patient-facing software interventions that can be tailored to individual's needs by combining prescribed medications with app-based systems. Since there is no need for physical logistics, DTx can complement the Decentralized Clinical Trials by allowing people access the healthcare services regardless of time and place. In future, the use of DTx will be widened by related technology such as cloud-rendered XR, single-digit millisecond latency with edge computing, and real-time predictive analytics (25, 107–111).

**Figure 6:**
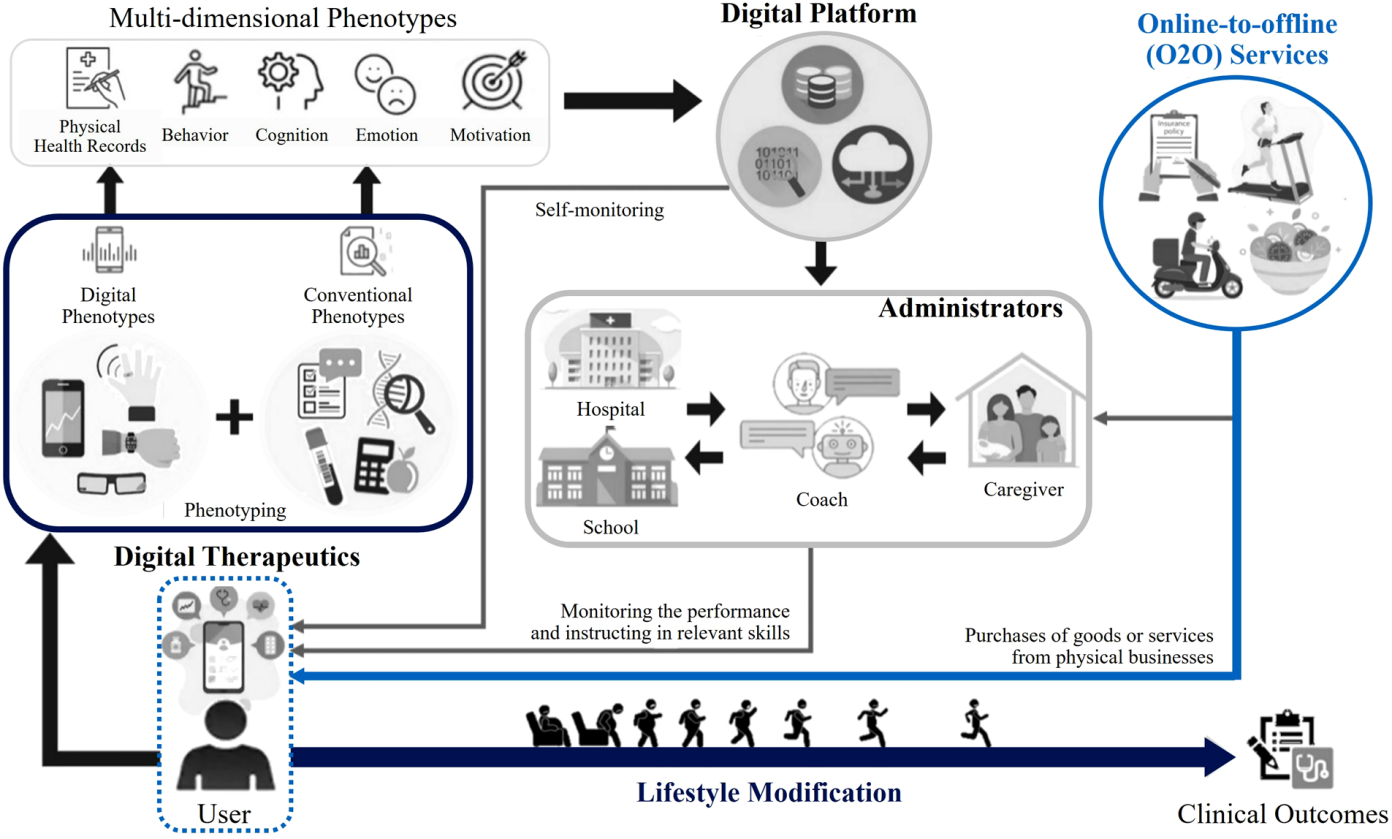
Dtx ecology for modifiable behaviors (112), showing future perspectives for the ecological environment of behavioral medicine that leverages digital technologies, including XR. Reproduced with permission of the copyright owner.

Such digitally enabled approaches should work in tandem with carefully designed physical infrastructure, urban space, and buildings to deliver truly preventive healthcare services and healthier lifestyles (89). As shown in Figure 6, the ecological environment of DTx has been proposed by medical researchers to support a sustainable business model of behavioral medicine for obesity and eating disorders. DTx can be combined with online-to-offline services within a ubiquitous environment of the future healthcare ecosystems (112). Here, the concept of creating “Enabling Spaces” such as green spaces and walkable communities can tackle the noncommunicable chronic diseases and modifiable lifestyle factors (113) by enhancing the efficacy of DTx, which is one of the key digital health modalities that require a comprehensive integration of technology and built environment.

The usability of DTx can be reinforced by MXR and Digital Twins (DTs). DTs can be used as the virtual representation of target patients’ physiology for *in silico* clinical trials. And the DTs of healthcare facilities can bring operational benefits such as monitoring bed shortages and optimizing staff schedules (114). Combined with Machine Learning and Artificial Intelligence, DTs continue to evolve in healthcare and life sciences to provide precision medicines as part of patient-centered care (115, 116). Eventually, MXR, DTx and DTs will become indispensable parts of future healthcare ecosystems. On top of that, the healthcare industry now seeks to enter a collective and incentivizing virtual environment, “the Metaverse”, which is accessible by immersive technologies. “Metaverse” is a portmanteau word formed from “Meta” and “Universe” and it has gained traction as a combinatorial innovation of flexible work styles and spatial technologies (27).

In 2021, Seoul National University Hospital gave lung cancer surgery training to the avatars of surgeons from around the world through the 360°-8K-3D metaverse platform. This was to meet the demand for non-face-to-face education during the COVID-19 pandemic (117). Also, South Korean's Metaverse Doctors Alliance (MDA) adopted its own cryptocurrency as payment method for the health services they provide. And the MDA's MetaClinic resembles the spatial layouts of real-world hospitals to give a sense of familiarity that is strongly associated with human cognition (118). In 2022, the US's Food and Drug Administration (FDA) has hosted its own MXR programs to approve key metaverse solutions (119). In particular, its economic profitability has been highlighted because the metaverse will dramatically transform the healthcare sector by innovating clinical education and patient care, but more importantly, introducing monetization through blockchain, gamification, and Non-Fungible Tokens (NFTs) (120).

Whilst admitting that in-person consultations in a shared immersive environment allow far more natural interactions and collaborative treatments (121), the cautious approach, on the contrary, warns about the risks of investing in many individual health metaverses. It is because the metaverse has limited functionality in its current form although a quarter of population will extend their physical activities to this digitally enhanced virtual world by 2026 (27). Thus, it can be challenging to acquire a complete picture of how the metaverse will alter the future landscape of health and care services. Nevertheless, there is one consensus among researchers and practitioners on the short-term outlook of healthcare metaverse; the future healthcare ecosystems need to strike the right balance of digital and in-person healthcare services (119, 122). In consideration of these points, the last hypothesis to be tested in relation to the third research question is as follows.

**Hypothesis 3**: With the rise of metaverse, XR will become a core component of digital health revolution to transform the way healthcare is practiced and delivered. More adaptive and inclusive approaches to the development of health-promoting environment are essential for built environment experts to embrace more decentralized, preventive, and therapeutic characteristics of the future healthcare ecosystems.

## 3. Methodology

### 3.1. Research design

The research is designed to understand the most agreed body of knowledge and different accounts of MXR and their implications on built environment. Since the amount of MXR-related data is currently limited, this research relies on two qualitative methodologies. An exploratory literature review was chosen to identify the variables of theoretical frameworks to assess the research questions. To create continuity while bridging a gap in existing studies, those theoretical frameworks were used to formulate hypotheses, one from each thematic analysis of related work, and provided directions for answering the research questions. Given the scarcity of research data, the purposes of interviews were to gather empirical evidence from participants who can provide scientific narratives into the situations, scenarios, and processes of MXR applications in the healthcare systems.

(i) Exploratory literature review with a critical evaluation of secondary data from the existing published work, including peer-reviewed academic journals; statistical data from governmental reports, private sector research and surveys; and edited academic and professional books.
(ii) Author's own observations on primary data collected from individual, semi-structured, and in-depth interviews with nine key informants, including industry experts and academic scholars with specialties and professional backgrounds relating to the immersive technologies in healthcare sector.

**Figure 7:**
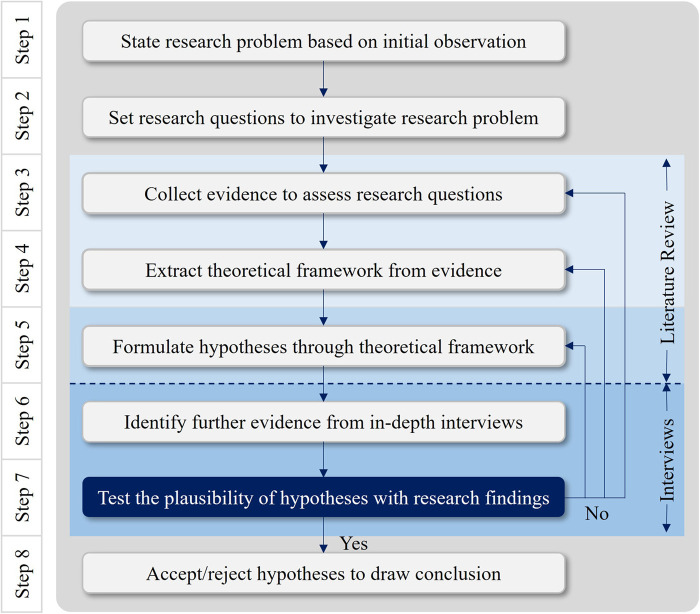
Qualitative research process.

Figure 7 depicts the non-linear and iterative process of implementing research findings to verify hypotheses.

### 3.2. Data collection procedures and participants

The research proposal has been approved by the School of Sustainable Construction Ethics Committee. Due to the restrictions concerning the COVID-19 pandemic, the author was advised to proceed with the online- and desk-based research methods rather than adopting face-to-face fieldwork or large-scale survey. Consequently, semi-structured and in-depth interviews were conducted online, using video conferencing (VC) and emails. The types of interviews depended on the availability of participants. All interviews were held in free-flowing settings to uncover meaningful patterns of describing how MXR alters the healthcare services and facilities and how built environment experts can cope with the future healthcare ecosystems transformed by MXR-related technological regimes. The researcher produced written notes and completed transcripts of video recordings to document the interviews. Findings were supplemented by follow-up questions to the participants. Sessions were carried out for 3 months, completed with the last one held on the 4th of August 2022.

During the video or email interviews, participants were given a list of open-ended questions within predetermined thematic frameworks. Researcher asked participants’ experiences, context-rich personal accounts, and forward-looking perspectives about: (1) Interviewee's expertise of MXR; (2) Risks and limitations experienced by the interviewee in MXR practices; (3) Interviewee's understanding on the relationship between MXR and built environment where the technology is embedded; (4) Interviewee's suggestions on how built environment experts can contribute to the implementation of MXR; and (5) Potential MXR applications in healthcare facilities the interviewee think it may enable better healthcare practices and outcomes. Table 3 summarizes the details of interviewees. The interviewees encompass great expertise in business, healthcare, architecture, computer science, and robotics engineering.

**Table 3:** List of interviewees.

ID	Date (dd/mm/yyyy)	Type	Field	Backgrounds
A	02.05.2022	Email	Business	Founder of a leading British company specialized in XR events for global audiences, B2B/B2C solutions, and VR surgery training
B	14.06.2022	VC	Business	Director of XR hub; Keynote speaker at international XR conferences; XR chair professor in British university
C	04.07.2022	Email	Healthcare	Founder of MXR alliance; Managing director in a leading AI-powered mental health platform company; Former technology director at NHS foundation trust
D	05.07.2022	VC	Architecture	Computer-aided design technician at NHS foundation trust; Freelance metaverse property developer
E	11.07.2022	VC	Computer Science	Deputy director of data science academy; Co-founder of a leading prototyping company; Computer science and information lecturer in British university
F	15.07.2022	VC	Architecture	Healthcare design director at a leading British construction company
G	25.07.2022	Email	Healthcare	Chair of medical metaverse research association; Neurosurgeon; Medical professor in South Korean university
H	29.07.2022	VC	Architecture	Founder of architectural practice designing virtual environment; Architecture professor in South Korean university
I	04.08.2022	Email	Computer Science	VR digital design accessibility researcher; HCI lecturer in British university

## 4. Results

### 4.1. Data characteristics

This research process presents qualitative findings by providing a statement of in-depth interviews with nine key informants. The original quotes and examples drawn from interview transcripts were compiled as raw data (see the Supplementary Material) before they were further analyzed. All interviewees remain anonymous to ensure their confidentiality and privacy. Supplemental evidence is provided to support the interviewees’ contributions in determining whether the phenomenon described in each hypothesis can be validated or nullified. As a result, the findings are classified into three thematic areas: (1) Synergism between Architecture and Technology; (2) Patient Empowerment and Staff Support; and (3) Scalable Health and Wellbeing in Non-hospital and Therapeutic Settings.

### 4.2. Synergism between architecture and technology

MXR will contribute to the resilient healthcare systems of the post-pandemic world by combining medical expertise with immersive experiences that go beyond the traditional healthcare provision. For the large-scale deployment of MXR, many researchers and practitioners have highlighted the importance of three practical methods: cross-sectoral initiatives; evidence-based design; and participatory approaches. Interviewee A (IA) is a founder of world-renowned XR company collaborating with various industry experts. In 2016, IA's company launched the first global VR-content distribution platform for medical training and education. IA advised, “If we investigate how XR can improve the treatment of stress or anxiety, there should be an explanation as to how this relates to the built environment.”

Although built environment experts have long been a part of cross-sectoral initiatives to create clinical environments where healthcare communities can grow and thrive, however they barely got a look in on “the Growing Value of XR in Healthcare” (16, 39). Yet, clarification is needed as to whether it is about the roles of built environment experts around the 3D visualization of space or their potential contribution to create physical environment that works best for the MXR implementation. A founder of MXR alliance, Interviewee C (IC) responded, “The built environment knowledge and expertise are inherent in the skills of the coders and designers. Those who built XR solutions have considered the mechanics of the built environment. However, I think the key here is ‘considered’.” IC then remarked that the active participation of architects will enhance the MXR applications further.

In architectural practice, XR is being increasingly used for the design and development of healthcare facilities. Interviewee F (IF), a healthcare design director of a leading construction company, shared the experience of using VR walkthroughs to help the client understand what a new hospital looks like. IF mentioned, “In many cases, the virtual tour is most effective when combined with full size mock-ups at 1:1 scale because people are familiar with neither two-dimensional drawings nor entirely virtual environment.” Interviewee H (IH), a virtual space architect, uses Immersive Virtual Environment (IVE) to simulate optimal lighting design for workplace. IH said, “The same applies to healthcare facilities as doctors also need offices, not to mention lighting as a critical element for hospital to promote the patient's wellbeing.”

Another important point is that built environment can ensure the best use of MXR. Interviewee I (II), a HCI researcher who develops an accessible virtual environment for visually impaired people, explained that the earliest headsets with extra cables and sensors posed a huge risk to users. II elaborated, “Any SteamVR solutions using base stations cannot have reflection in the space. Lighting conditions must be accurate, and normally these must be placed in elevated positions for accurate tracking.” Even the most advanced HMDs can be susceptible to the level of “Neutrality” in test sites. II added, “All kinds of inside-out tracking HMDs should not be used outdoor or in contact with sunlight due to the risk of camera damage.” II was asked if he felt the need to collaborate with built environment experts. “To a certain level yes. When working with business I had to ensure that rooms were suitable.”

Conversely, built environment experts can leverage MXR's data-handling capacity for the evidence-based design of healthcare facilities. In the MXR landscape, tracking sensor technology can decide the usability of MXR as to the impact of physical environment on digital world because it can collect and process enormous data about the users and their surroundings (79). Interviewee E (IE), a computer science and informatics professor in British university, described how useful these sensors can be in elucidating the relations in between people's health, their lifestyles, and built environment. “We can think of a physical environment which lets the robot and sensors function seamlessly and doesn't get in its way.” So, one could infer from this statement that the data collected from MXR sensors can be the basis of architectural design to aid people with disabilities or disorders.

IE also urged, “Knowing what technologies are needed for the house is a big thing. I think that's where the key is for. We have all these studies being done in the house for healthy aging or People with Dementia (PwD). But I don't think anybody has seriously taken this.” In fact, the XR utilization via IoT platforms has been developed under the umbrella concept, “Smart Home” or “Smart Cities” to enhance the elderly's autonomy and independence in their lives (123, 124). Unfortunately, current practices remain at retrofitting guidelines for general homes rather than being specified for living spaces with extensible technology adoption (125). On a somewhat related note, Interviewee I proposed, “Healthcare facilities need to involve tech experts who can suggest solutions to current procedures and practices that would save a lot of time and money, and greater awareness is needed to achieve this.”

**Figure 8:**
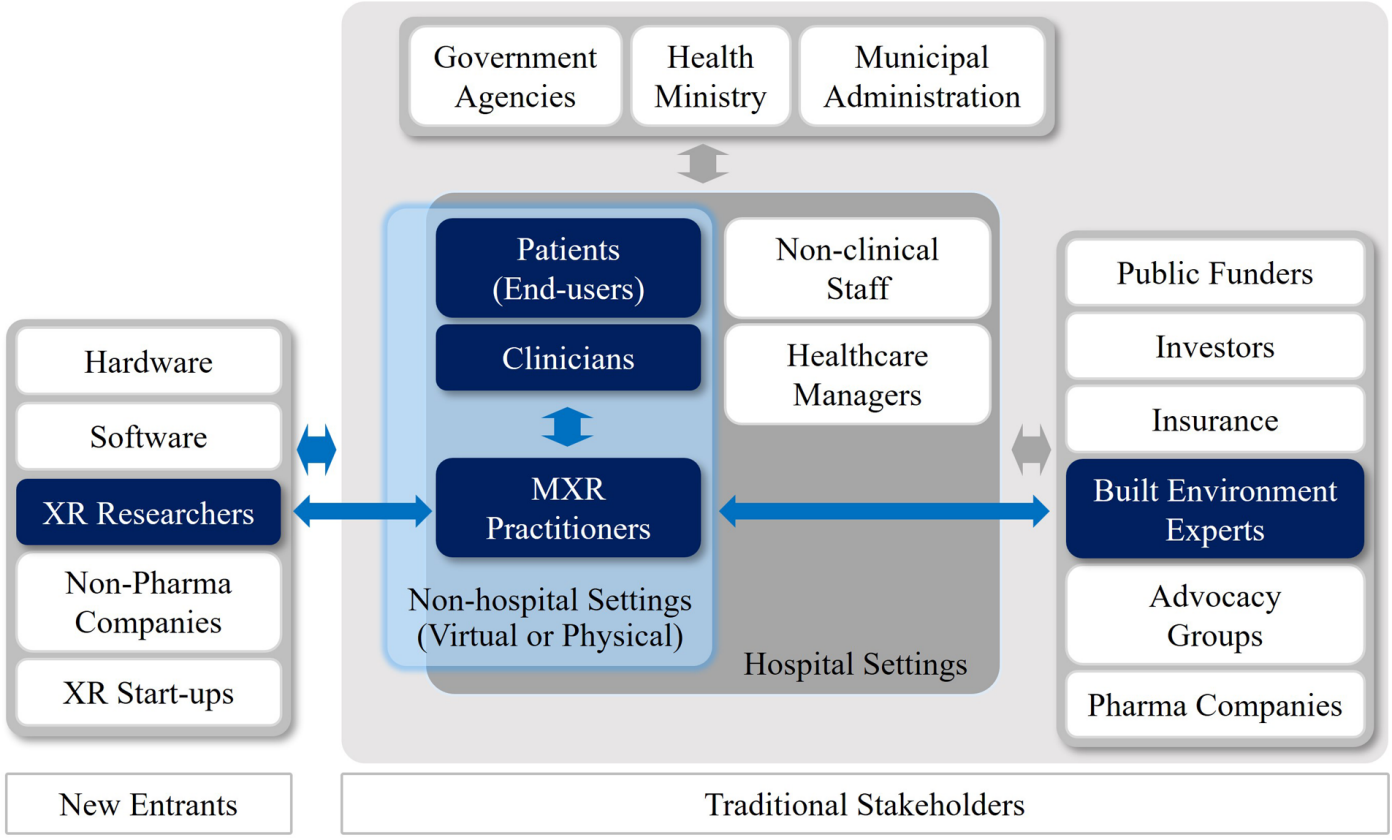
Stakeholders in MXR ecosystems.

Interviewee F (IF), an architect, was asked about the leadership potential and communication ability of built environment experts in engaging with a disparate group of stakeholders. IF replied, “Architects are only very small part of complex and extensive healthcare systems. Moreover, stakeholder dynamics and project requirements will vary depending on the programs and stages of healthcare facilities projects.” For this reason, many MXR pioneers advocated a representative and impartial network of academic institutions, researchers, healthcare providers, digital and creative industries to share skills and resources, while avoiding duplications (126). Figure 8 illustrates the author's interpretation regarding the different domains and cross areas of traditional stakeholders and new entrants in the MXR ecosystems.

As mentioned earlier, Participatory Design (PD) is one of key elements embedded in healthcare ecosystems. Interviewee F elucidated that PD is not just necessary to cater for the different needs of stakeholders in the healthcare ecosystems, but also mandatory to get funding for most healthcare facilities projects in the UK. Interviewee B, a chair professor of XR in British university and a keynote speaker of numerous international XR conferences, expanded on this point, “When it comes to the alteration of built environment for XR implementation, it is important to understand multifarious demands from different stakeholders.” Specifically, before a comprehensive MXR infrastructure can be built, the targeted use cases and intended Return on Investment (ROI) must be qualified over both time and intent. And benchmark must be outlined (63).

### 4.3. Patient empowerment and staff support

No matter how amazing health innovation may be, its long-term integration into the systems often comes down to basic user experience. XR enables the quality of care by lowering the physical impact on the patient, shortening their recovery time, and therefore resulting in significant healthcare savings (63). Also, XR strengthens humanity in healthcare with an inner-centered conception of patient's life (85). Interviewee I (II) shared an idea on this, “From years of collaborating with impaired users, I have seen first-hand that XR can bring dramatic changes to someone's everyday life. However, XR is heavily underutilized for these kinds of practical implementations.” II continued, “VR devices could be used as highly advanced visual accessibility aids to replace several existing tools and equipment that are relied on currently at a fraction of the cost.” In other words, the XR-enabled healthcare transformation should be understood in the context of far-reaching implications of the shift to more patient-centered and value-based health and social care.

The essence of MXR is the user experiences that can be enhanced through more engaging dialogues between patients and healthcare providers, while allowing clinicians to focus on more urgent and chronic health issues. Working with the NHS on specifying the user requirements of VR anxiety and depression treatment, Interviewee B emphasized, “The most desirable health technology is not the kind of cutting-edge ones. Clinicians welcome a novel digital tool to save cost and time, to get better clinical outcomes, and to make patients satisfied. If technology fails to make a huge improvement compared to traditional health practices, it will get rejected very quickly. Cost and performance are the critical factors.”

That being said, it would be remiss of us to neglect the XR's practical problems, including laborious regulations, licensing restrictions, lack of rewarding payment methods, clinically undeveloped technologies, and patient confidentiality regarding health data (127, 128). By adopting Gartner's well-known Hype Cycles of Digital Health, there are five distinct levels to describe the maturity of XR-related digital health technologies. Especially, the rigor of evidence-based decision-making founded on well-constructed trials and data supporting best practice are essential to escape the “Trough of Disillusionment” (128). Interviewee B indicated, “The current Hype of digital health technology is positive in terms of attracting investment for research going forward. But there are always very conservative people who are reluctant to decide investment before they see concrete evidence.”

Therefore, a better understanding of clinical workflows, patient adherence characteristics, behavioral interventions, and more importantly getting buy-in from both clinicians and patients are business imperatives to widespread MXR (4, 127–130). Interviewee B highlighted, “Marketizing XR requires a huge investment to train and educate people to adapt to new digital health technologies. Some serious investment decision will only be made on firm evidence. It will then finally lead to the alteration of physical environment to accommodate any new requirements.” Particularly, patient engagement is a critical factor when determining the success of the XR-related interventions (63). For this reason, XR is used as the reproduction of human body or healthcare facility to familiarize the patients with clinical environments or procedures, thus making patients feel more empowered. The examples are as follows.

In the US, Stanford Virtual Heart pioneered XR to help physicians treat cardiac patients more effectively by informing people of the most complex congenital health problems (131). The Hoag Centre for Advanced Visualization and Immersive Therapeutics (AVIT) in California uses patient-specific 360° models for patient engagement, pre-operative planning, and surgical rehearsal (130). In the UK, the Dental Public Health at the Wales University Dental Hospital (UDH) augmented patient's experience with virtual characters and gamification (132). Interviewee E (IE) worked on this project in close liaison with pediatric dentistry, public health consultants, digital learning managers, and software developers. IE commented, “We gave children a virtual tour by putting virtual characters who can speak to children so that they can get familiar with the environment of dental clinic. At least, children have one less thing to worry about.” This was indeed a good co-production of virtual and physical healthcare facilities to demystify dental visits by increasing patient's confidence levels, thus making patient care easier for clinicians.

**Figure 9:**
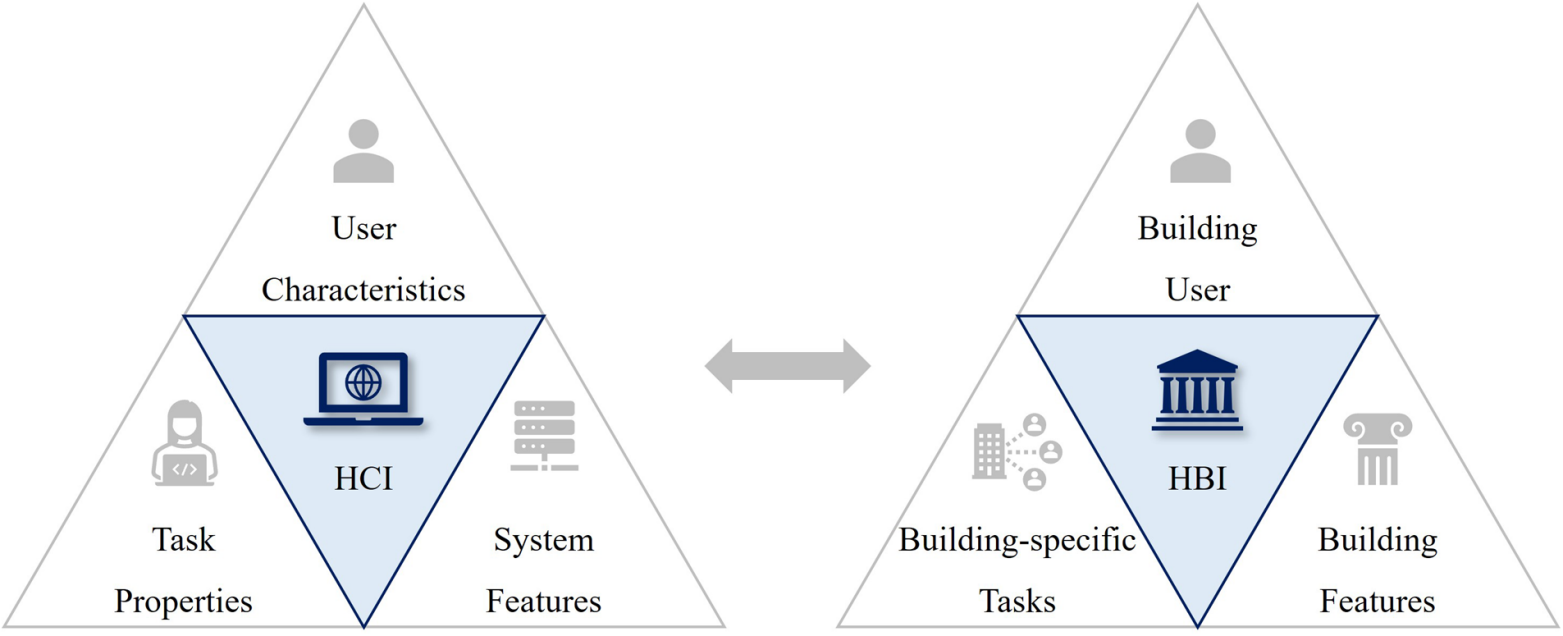
Key usability elements in HCI and HBI, comparing three elements (133) that constitute the definition of usability in human-computer interactions and human-building interactions.

Regarding the user experiences, another important aspect to consider is HCI which is the XR's operation principle, enabling interactive experiences in an immersive environment. Interviewee I (II), a HCI scientist, was asked about the notion of HCI in relation to the built environment. II answered, “Since some testing was done comparatively to physical elements, so a recreation of the built environment was necessary. This considered variables such as the luminosity levels and distance. To avoid the need of physical movement, distances could be manually manipulated in a controlled scenario that was a replication of the actual built environment.” This appears like the way HCI is translated into HBI by architects as a theoretical framework to understand “Usability” and “User-centric Experience”. In other words, both architect and HCI scientist have the same interpretation that “usability is ultimately about avoiding frustrating the user” (133) (see Figure 9).

To avoid unwanted side-effects of MXR, one critical solution is to emulate what goes on in the real world. Interview G, a neurosurgeon who chairs the medical metaverse association in South Korean university, stated, “A virtual clinic should be designed following the layouts of actual consulting, examination, and treatment rooms because they can become the deciding factors affecting patients’ mental and emotional health.” Interviewee H (IH), an architect, designed the virtual offices for a South Korean telecommunication company. IH mentioned, “After a long discussion with our client, we decided to provide people with a familiar place rather than making it look so different from its real-life counterpart. The general design standards we've been using for physical environment work well with our spatial cognition because they are proven with certainty through the history of architecture.” This could imply that architects should become more involved in, not just the coordination of physical settings to ensure MXR's safety, but also the design of virtual environment itself.

### 4.4. Scalable health and wellbeing in non-hospital and therapeutic settings

Digital disruption would generate new entrants, new business models and new locations (101). The amplified need for remote care during the periods of COVID-19 quarantine and isolation has led to new business models such as the digital apps, providing significant health benefits and commercial success (134). Likewise, XR could easily be the greatest industry disruption healthcare has seen in over one hundred years (63). As a computer-aided design technician at NHS foundation trust, Interviewee D (ID) said, “We can learn from how e-commerce changed the industry. They keep most of their logistics facilities. The healthcare sector will use Urban Air Mobility (UAM) to reduce the cost and time associated with medical logistics. Small and easy tasks can be delivered virtually. But large hospitals will always need to keep high-tech and heavy medical devices.” Additionally, ID suggested that there will be more user-centric programs in the future hospitals, as digital and logistics technologies advance, just like many e-commerce business models now focus on customer experiences.

Certainly, some healthcare infrastructure will migrate from the status of *in vivo* to *in silico*. Henceforth, the future healthcare ecosystems will have more decentralized, preventive, and therapeutic characteristics. Interviewee B commented, “XR extends health-promoting spaces and opportunities for more people. Currently, healthcare providers encourage people to come to the medical center or do it online. If people do not come, it will incur a cost anyway. Hospitalization and the expense of inpatient treatment are astronomical from the government's perspective. That is why preventive care is so important. Government wants to keep people healthy.” Therefore, more adaptive, and inclusive approaches should be taken by built environment experts to embrace those characteristics in developing a health-promoting environment. This could range from introducing an exclusive space to ensure the best use of MXR to redefining the relationships between existing and new healthcare premises.

Interviewee B (IB) has been working with the NHS on several mental health projects, including the Pulmonary Rehabilitation in Virtual Reality (PRinVR) programs for more than three hundred lung cancer patients. IB replied to the interview question of whether we can decide a certain kind of healthcare service is more suitable for virtual migration than the other. “It will vary across the departments. The issue is greatly affected by the requirements, financial status, and future directions of each hospital department.” IB stressed that it will all boil down to a question of cost saving as well as better clinical outcome. Interviewee F, a very experienced healthcare architect, forecasted, “We may end up with less clinical spaces, more therapeutic spaces. In the hospital, there will be more soft spaces which can be easily relocated in comparatively low cost.” Also, there is a prediction on the percentage shift of health and care services that ripe for significant virtual health migration by 2040, which will accelerate such transformational changes (127).

In the US, many healthcare providers start reimagining the future healthcare built environment to be designed around primary and community care (129). The aim is to coordinate all health-related issues by multidisciplinary teams in readiness for the transition of healthcare systems and services. Focusing on the prediction and prevention of diseases, advances in digital health will shift both operations and leadership from hospital-level care to the home or community surgical centers. And chronic disease management programs that are not anchored to a hospital or clinic will be more taken by MXR. Meanwhile, a visionary model for the optimization of health resources was awarded the 2021 Wolfson Economics Prize in the UK. This work suggests that XR will enable the healthcare industry to overcome geographical restrictions, future hospitals will exist as virtual space with physical outposts in community locations. Consequently, only the services that cannot be digitized will remain at the large acute hospitals (135, 136).

Such projections are in line with the central aim of ICSs to join up hospitals and community-based services as partnerships across different organizations and settings (137, 138). Similarly, Arup proposed that future healthcare ecosystems will be “smaller, better connected, and strategically dispersed in cities, with a stronger local presence and identity”. A better data connectivity and interoperability will enable these alternative healthcare facilities to implement more domestic, natural, and therapeutic elements for bespoke patient experiences by accommodating new lifestyles and demographic factors. And eventually, they will challenge the design conventions of traditional healthcare premises (89).

Interviewee E, a robotic scientist, shared the vision of future built environment from an engineering point of view. “The more you overcrowd, the less ability you give any technology. Anything moving around and you're looking around, you can crash into things that are less space free to move around. This comes from sci-fi, there's sort of truth in what they build. Robotics and everything require a kind of clean, smooth, and quite minimalistic environment. You need a lot more minimalistic environment in a big space.” This could imply that emerging technologies such as XR involving IoT sensors, will not only become useful tools for healthcare services, but also pave the way for future healthcare ecosystems that necessitate a change of concept in designing healthcare facilities.

Interviewee D (ID) is also a freelance metaverse property developer. When it comes to the design of healthcare facilities in metaverse, ID said, “Architects can have more freedom to design, invest, and develop some cool experiences for people as we understand what the human-centered design is. This is the way I perceive the virtual environment; we are shaping the experiences in it.” Opinions diverged greatly on the design of virtual clinics. A neurosurgeon, Interview G mentioned, “The medical metaverse refers to not just a visually experiential space, but also a new spatiotemporal layer in which Clinician-Patient, Clinician-Clinician, and Trainer-Trainee can communicate with each other. The concept of medical metaverse should be a more profound yet practical version than ones for games or entertainment. In that sense, the use of avatar or spatial layouts are not the critical issues.” In the US, Cannon Design proposed “Virtual Care Modules” as mission control centers for clinicians to make virtual visits as a preparation for a shift from in-person to virtual care. In parallel, physical facilities will need digital pods for one-on-one sessions and larger spaces for the care teams to replicate virtual and physical activities (139).

## 5. Discussion

### 5.1. Key findings

#### 5.1.1. Synergism between architecture and technology

The first research question was about the opportunities presented by MXR and whether built environment experts can benefit from them. This leads to the first hypothesis that the synergism between architecture and technology will take on added significance as some healthcare resources will migrate from the status of *in vivo* to *in silico*. The process involves cross-sectoral initiatives, evidence-based design, and participatory approaches, which have long been the common denominators of two disciplines. The reproducible, replaceable, and interactive characteristics of XR can reinforce the provision of health and social care, especially when its scalability is imperative amid public health crises. During the research, XR-enabled advantages for built environment experts were readily identifiable as to the development of healthcare facilities. In contrast, their potential contributions to the advancement of MXR for post-pandemic resilience have been largely overlooked.

**Figure 10:**
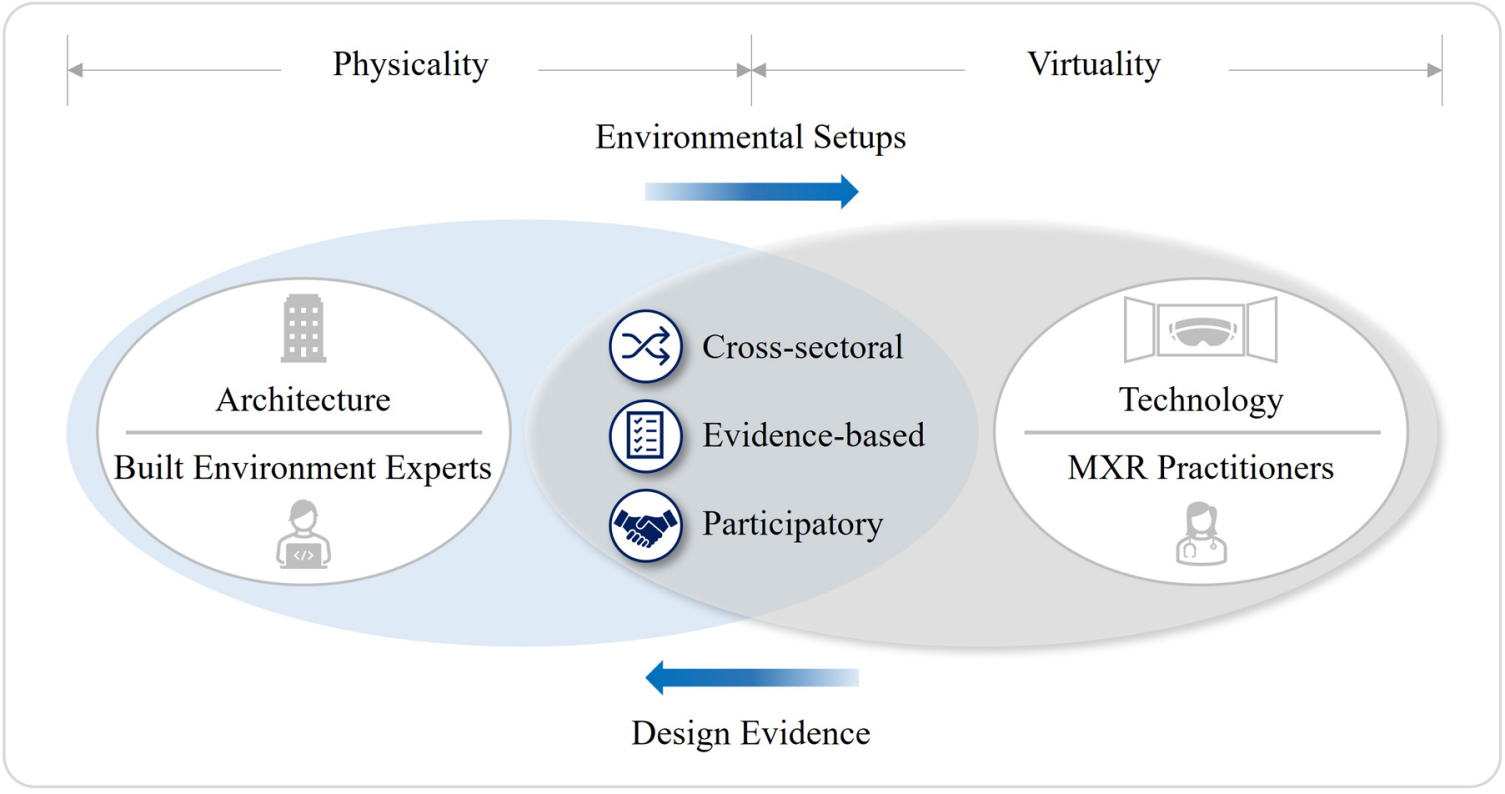
1st hypothesis verification.

This could be because the MXR ecosystems consist mainly of healthcare providers, software/hardware engineers, and digital content creators. The other reason is that MXR practitioners generally lack understanding of how built environment experts can underpin the operation of MXR. Research participants shed light on this reciprocal relationship between MXR and the built environment. For better patient experiences and health outcomes, the current modality of MXR applications needs to keep certain environmental factors under control. This necessitates architectural interventions to ensure the efficacy of MXR. Also, the analysis shows that leveraging the capacity of MXR for collecting user-specific health and lifestyle data can complement the evidence-based design of any health-promoting built environment including healthcare facilities. For these reasons, it would benefit both MXR and built environment communities to raise awareness towards the need of more coherent framework for participatory approaches to cater for the different demands of stakeholders (see Figure 10).

#### 5.1.2. Patient empowerment and staff support

The second research question investigated the challenges posed by MXR and whether built environment experts can alleviate those problems. This has been made into the second hypothesis that a collective effort by MXR and built environment communities can increase the interoperability and usability of MXR as the next-gen digital medicine. Whilst the exploratory literature review focused on collecting evidence of administrative complexities or technical huddles to the MXR implementation, the qualitative data analysis of interviews specified practical problems where the practical skill sets of built environment experts can be used appropriately, and ultimately how it can facilitate the patient-centered and value-based models of health and social care. As a result, patient empowerment and staff support through user-centered experiences were identified as the ultimate purposes of MXR.

**Figure 11:**
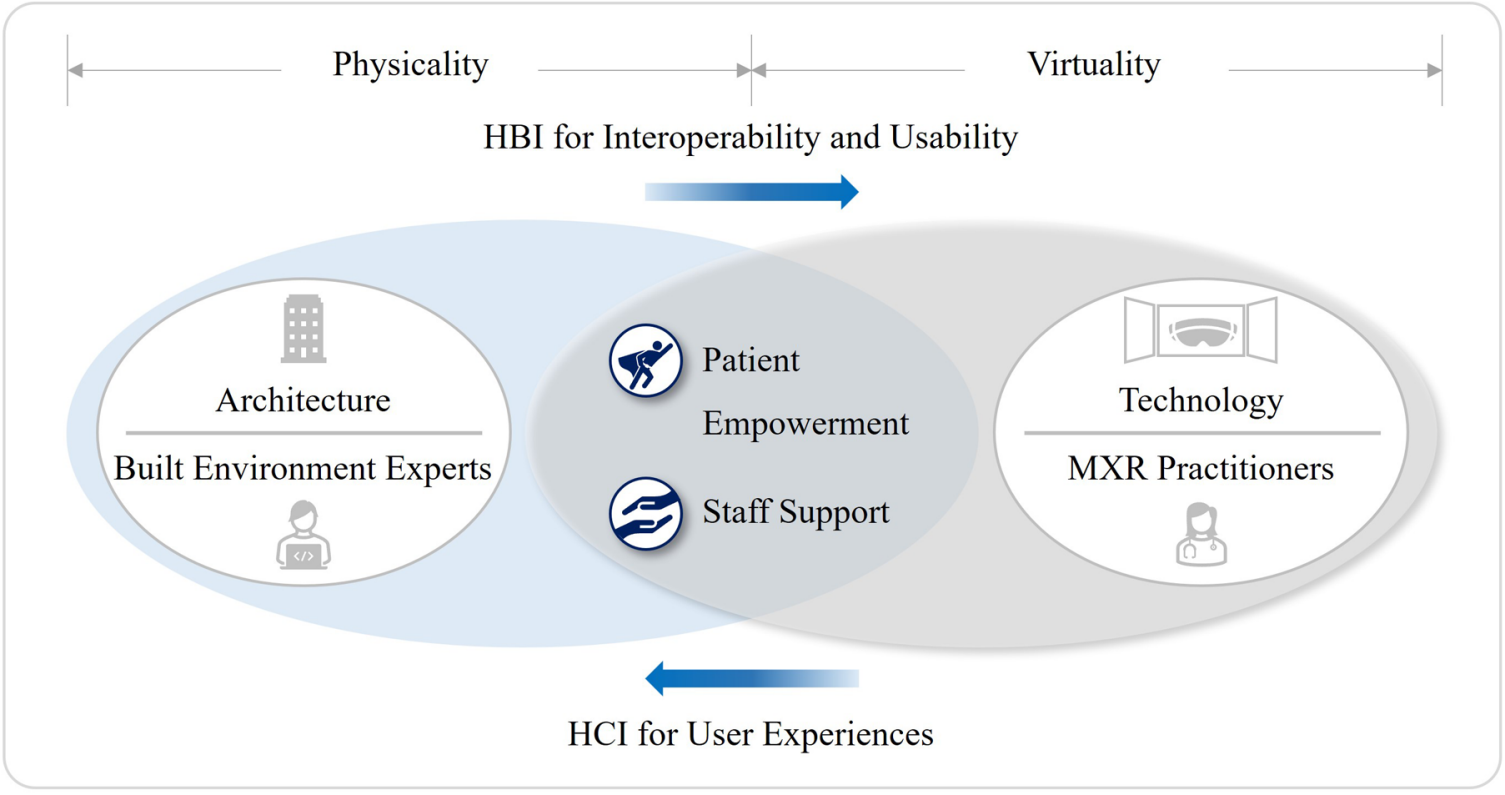
2nd hypothesis verification.

It was clear from case studies that XR as the reproduction of human body or healthcare facility can effectively increase the patients’ understanding along the medical procedures, thus making them less burdensome for the clinicians. System requirements and user concerns are two key aspects to understand how HCI enables XR's multi-sensory experience in virtual environment. Among the XR's therapeutic mechanisms, strengthening patient's self-identity through HCI can significantly improve the usability of MXR. Since architects have rigorously studied HCI as to the user-centered experiences, a confluence of knowledge and methods on HCI will benefit both MXR and built environment sectors. Additionally, XR needs to mimic reality as much as it can to prevent the occurrence of any unwanted side-effects such as “Cybersickness”. In this regard, the analysis suggests that the ability of built environment experts, especially architects, can be extended to the creation of virtual environment by bringing their expertise on spatial organization and its implications on human physiology and psychology (see Figure 11).

#### 5.1.3. Scalable health and wellbeing in non-hospital and therapeutic settings

The third research question was to examine the roles and responsibilities of built environment experts in constructing a long-standing value chain of healthcare premises that will be transformed by the nascent XR regime. The third hypothesis posits that XR will foster more decentralized, preventive, and therapeutic characteristics in the future healthcare ecosystems. Since each characteristic would induce the alteration of physical settings, built environment experts should take more adaptive and inclusive approaches to the planning and design of healthcare facilities. XR-enabled digital disruption will accelerate decentralized healthcare services by spatially dispersed and specialized facilities with a strong local presence. Also, there will be a “Virtual Migration” of some healthcare services just as we have seen how e-commerce reshaped the real estate industry during the COVID-19 pandemic. Further analysis, however, revealed that the healthcare industry will not exactly follow the same pattern because in-person consultations and in-patient treatments at the large acute hospitals will still be indispensable.

**Figure 12:**
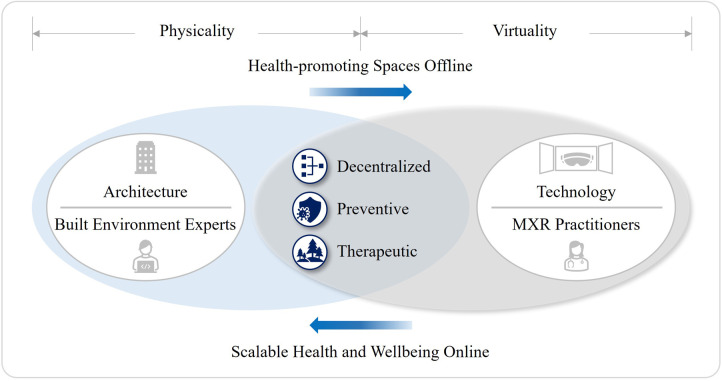
3rd hypothesis verification.

Additionally, XR will strengthen the efficacy of DTx which offers precise and preventive medicine. As part of the treatment, DTx requires the so-called “Enabling Spaces” for promoting active and healthy lifestyles. Hence, built environment experts should support the future healthcare systems by creating more therapeutic architecture to invigorate individual's wellbeing as opposed to the conventional design of existing healthcare facilities. Lastly, the data suggested that XR-related technologies such as IoT sensors and UAM will also affect the way healthcare premises are designed. Regarding a novel concept of “Healthcare Metaverse”, it was identified that the balance of digital and in-person healthcare services for more efficient clinical workflows will become a critical matter as to the development of virtual clinics or healthcare platforms in metaverse (see Figure 12).

### 5.2. Research gaps and limitations

Recently, the UN's Sustainable Development Goal (SDGs), the Environmental, Social and Governance (ESG) commitments, and the large-scale digitization have become three megatrends to evaluate investment opportunities for the post-COVID economic recovery. The study would therefore have benefitted from the XR's sustainability aspects in investigating how a long-standing value chain of healthcare premises can be generated in relation to the MXR-induced disruption. Although it is presumed that MXR could be part of a circular economy for the sustainable growth of healthcare industry, however this aspect remains unverifiable at present due to the lack of empirical data concerning MXR-related consumption and production strategies for energy, labor, and materials.

In addition, qualitative data was collected from a relatively small sample size. This would mean that research quality is somewhat dependent on participant's different set of values and expectations about the present state and future prospect of MXR. Thus, further evidence addressing the long-term implications of MXR on both healthcare and built environment systems would be required to generalize the findings beyond the research boundaries.

## 6. Conclusion

This qualitative research has strived to examine the implications of XR on healthcare systems and built environments. Also, the research has identified how built environment experts can manage the XR-enabled innovation for healthcare in a more structured and deliberate way. Since most healthcare capital projects take considerable time to complete, a pre-emptive approach should be taken to the wide-scale implementation of XR so that the healthcare systems can ensure resilience in a post-pandemic future. Three questions have been answered through the process of implementing research findings to verify hypotheses.

Firstly, despite an inadequate recognition of built environment experts’ potential contribution, both built environment and healthcare sectors can benefit from the synergism between architecture and technology by leveraging the various capabilities of XR through cross-sectoral initiatives, evidence-based practices, and participatory approaches. Secondly, a confluence of knowledge and methods of two sectors can improve patient empowerment and staff support through two theoretical frameworks: Human-Computer and Human-Building interactions. The increased interoperability and usability of XR as the next-generation medicine will enhance the patient-centered and value-based models of health and social care. Thirdly and lastly, the XR-enabled technological regime including healthcare metaverse will largely affect the forms of value in healthcare premises by fostering more decentralized, preventive, and therapeutic characteristics in the future healthcare ecosystems. Therefore, more adaptive, and inclusive approaches should be taken by built environment experts for the creation of a health-promoting environment in contrast to the conventional design language of healthcare facilities.

After all, this is ultimately about how to improve resource efficiency in the healthcare sector as a preparation for a paradigm shift caused by using XR. That is to say, the transition of health resources from the status of *in vivo* to *in silico* should be factored into the process of establishing healthcare infrastructure which takes enormous time and concerted effort. To conclude, the following suggestions are made for XR-related research and practice hereafter in both healthcare and built environment sectors.

(i) Only a little has been written on the XR's sustainability, relying on a conjecture that its immateriality and reproducibility helps control pollution by reducing waste and encouraging recycling. To be exact, the intersection of intangible services promoting clinical efficiency and tangible assets ensuring therapeutic environment is where MXR can flourish. Just as a circular economy culture has been integrated into the development of Net-Zero hospitals, XR-enabled technological regimes should also take sustainability-oriented approaches to seek inter-sectoral solutions for a “Clean Growth” of MXR.
(ii) Some argue that the skill sets of built environment experts will be seriously devalued and undermined in the virtual world that defies the law of physics and spatial design principles in the real world. However, this would hardly be the case for their roles in reinforcing the efficacy of MXR because: (1) Even highly digitized healthcare systems will still require the “Offline Networks of Therapeutic Environment”; and (2) Whether it is virtual or physical, our healthcare systems have placed great emphasis on the rigor of evidence-based approach linking health outcome to a clinical environment. Henceforth, built environment experts should seek closer ties with the MXR ecosystems to make a real business case for the “Co-production” of scalable health and wellbeing in non-hospital and therapeutic settings.

## Data Availability

The original contributions presented in the study are included in the article/**Supplementary Material**, further inquiries can be directed to the corresponding author.
