# Supplementary material for: Implications of immersive technologies in healthcare sector and its built environment
Source: Front Med Technol. 2023 Sep 20;5:1184925. doi: 10.3389/fmedt.2023.1184925 (PMC10548380; doi:10.3389/fmedt.2023.1184925)
Supplement: Supplementary file 1 [file Datasheet1.pdf]

Author's Note:

Below is a set of primary data that was extracted from semi-structured and in-depth interviews, which were part of qualitative research for the MSc dissertation completed in 2022. This document has been submitted as an additional file to the Frontiers in Medical Technology for the publication of an original research.

Supplementary Material. Interview Transcripts (Yang, 2022)

RS=Researcher, **I(X)**=Interviewee X

ID	Date (dd.mm.yyyy)	Type	Field	Duration	Notes/Transcripts
A	02.05.2022	Email	Business	N/A	N

RS: (The researcher discussed with the interviewee A about the research topic and questions)

IA: The third research question introduces the Built Environment and its professionals, but I feel that there needs to be a connection between the previous two questions and this one. For example, if we investigate how immersive technologies can improve the treatment of stress or anxiety, there needs to be a connection to how this will influence the built environment and its development.

IA: The methodology you propose is very solid but again, I would have liked the investigation not to be limited to discussing the uses of immersive technologies in the healthcare sector, but also some reference to how the built environment may be affected should be investigated. So, you might need to extend the list of contacts, to professionals in the built environment itself.

IA: You have an extensive literature review although, again, I would have prepared to see references more related to the application of XR in the actual sector of built environment rather than its applications to the other areas of health and medicine. There may not be a lot of such references relevant to healthcare facilities projects but there must be a lot referring to other types of built environments, general architecture, special purpose buildings and the whole design and development activities.

IA: In your case, treating dementia or reducing pain may not be relevant and more relevant could be applications that support the design and perhaps assessment through XR simulations of industrial or other special purpose buildings. There is so much information out there relating to immersive technologies and their applications and if you focus not so much on the medical and health-related applications (for which there have been numerous studies and publications), but instead focus on the built environment and how immersive technologies could affect its future, you may have a very original body of work which could easily (if you so wish) be extended into a PhD study, on the basis of originality and lack of similar prior art.

ID	Date (dd.mm.yyyy)	Type	Field	Duration	Notes/Transcripts
B	14.06.2022	VC	Business	00:43:30	T

RS: Can you explain your recent work relating to the medical XR?

IB: I am currently working on several mental health projects in both UK and South Korea. It is one of the most talked-about issue these days. We are at the early stage of developing user requirements for an anti-anxiety treatment. I am also evaluating the VR programmes that are already in use. With the NHS, we have developed the Pulmonary Rehabilitation in Virtual Reality (PRinVR) programme for more than 300 lung cancer patients and monitored user experiences and feedbacks. I have experienced a wide range of medical XR applications. The use of telemedicine has been legalised in US and UK, but I have not seen many end-user cases of XR that have reached a full commercialisation yet.

RS: How long do you think it will take for MXR to become common practice?

IB: It would take a quite a long time. It depends on whether the application needs to be approved by the government agency such as the FDA in US or the KFDA in South Korea. And that is why many health-related or pharmaceutical company take so called ‘soft’ approach to dealing with either health or wellbeing. Clinical trials are very demanding processes. Normally, it takes a minimum of 3-5 years to get the product commercialised.

RS: How about the governance?

IB: Data governance is extremely strict in the UK. The private sector tends to be more flexible than the NHS.

RS: From my understanding, the maturity of devices seems to be the most important aspect for XR applications. But do you see the need for any requirements relating to the built environment?

IB: It depends on the purpose of XR applications. The question is whether we want to go entirely virtual or not. Now, most XR applications are the hybrid of virtual and physical environment. Basically, XR is the extended version of our physical space and time. And the virtual environment exists within the physical world. In that sense, it can be a question of how we balance the needs of virtual and physical elements within the XR applications. Then, it might be critical to decide what to remain in physical world and what to become virtual. And if we apply this to the hospital space, the reallocation strategies of physical spaces and resources will vary depending on the needs of each clinical department. Such a trend will become a far-reaching phenomenon. Thank about the benefits of online learning although we are now returning to face-to-face settings for education. The online gaming industry hosts a large group of offline audiences in a huge space using the large screen for greater sense of engagement. Likewise, XR has enabled this hybrid format in various sectors. It can be virtual hospitals or cloud-based clinical services. This will lead to a huge variety of case studies regarding which particular clinicians or patients' group can benefit more from certain physical or virtual settings. Again, the issue of how to balance them will be greatly affected by the requirements, financial status, and future directions of each department in hospital.

RS: I agree.

IB: Ultimately, it will all boil down to a question of cost savings as well as better clinical outcomes. We need to be able to prove that XR is better than traditional methods in terms of cost and time savings. Getting the greatest 'bang for the buck' is critical to marketize XR requires a huge investment to train and educate people to adapt to new digital health technologies. Some will be convinced only by firm evidence, some will be willing to test XR even before they see the evidence, it depends. But one thing for sure is that some serious investment decision will only be made on firm evidence. Then it will then finally lead to the alterations of physical environment to accommodate any new requirements of XR.

RS: Speaking of the evidence, I noticed there is a lot of new investment coming into this field despite a shortfall of current research and knowledge.

IB: If we look on the bright side, the current 'Hype' of digital health technology is positive in terms of attracting investment for research going forward. But there are always very conservative people who are reluctant to decide before they see concrete evidence for investment.

RS: I assume that usually refers to healthcare providers?

IB: Usually, the most desirable health technology is not the kind of cutting-edge ones. Clinicians welcome a novel digital tool when it, saves cost and time; gets better clinical outcomes; and makes patients satisfied. If the technology fails to make a huge improvement compared to traditional health practices, it will get turned down very quickly. Doctors are busy. Cost and performance are the critical factors. From the built environment perspectives, a variety of needs from various stakeholders should be accommodated into the spatial layout. So, the most optimised way to utilise office, retails, residential and healthcare facilities wherever the XR technology will be embedded.

RS: I guess the pandemic has forced people to take innovations in a way?

IB: Yes, it is because people were left with virtually no options. Another common mistake I witnessed, especially in South Korea, the development of many healthcare technologies is led by the government. Many of them lack proven medical benefits and it goes obsolete very quickly. When it comes to the alteration of built environment for XR implementation, it is important to understand multifarious demands from different stakeholders and sectors.

RS: I found that XR can increase the access to healthcare services.

IB: Basically, XR extends the health-promoting space and opportunities for more people. The patient engagement rate of actual healthcare services is very low, which is less than 10%. Normally, healthcare providers encourage people to come to the medical centre or do it online. If people do not come, it will incur a cost anyway. Hospitalisation and the expense of inpatient treatment are astronomical from the government's perspective. That's why preventive care is so important. Government wants to keep people healthy. XR can be a great opportunity for medical doctors. The global market size for the medical XR is enormous.

IB: By the way, why did you choose this research subject?

RS: XR can be used for staff relaxation. And I originally wanted to investigate the various themes of XR contents for staff relaxation as retention strategy in the post-pandemic.

IB: Sure. The heavy workload of medical staff is very serious problem. There is a lot of information out there. Again, mental health in general is very critical topic in the UK.

RS: How would XR affect the healthcare facilities from property asset management perspective?

IB: If you want to know about how XR might affect the management of property assets, you can check what happened in other industries. Industry is always much faster than academia.

ID	Date (dd.mm.yyyy)	Type	Field	Duration	Notes/Transcripts
C	04.07.2022	Email	Healthcare	N/A	T

RS: You have established the medical XR alliance in the UK. Do your initiatives calling for multi-disciplinary effort infer the potential contribution of built environment experts as well?

IC: In what way would you envisage built environment specialist contributing to the XR health movement? Are you thinking just in general around 3D space visualisation and how to best create an environment that works? Or something else?

RS: My observation is that the current discourse on XR in healthcare is primarily led by either healthcare providers or devices/contents creators. In addition, the partnership mapped out by your XR alliance does not indicate the possible involvement of any built environment specialist yet. I assumed that architects could play the potential role in creating a 'sense of presence' within the AR/VR contents by mimicking the principles of actual clinical spaces such as furniture layouts, illuminance, or adjacency between the departments. And this would not only just help patient feel more comfortable with the VR sessions, but also help clinicians remain familiar and confident with their virtual workplaces, thus leading to more efficient medical practices and health outcomes. Another thought is that built environment experts should prepare for the future healthcare ecosystems where XR finally becomes an integral part of

healthcare services as this would require the reallocation or rearrangement of current healthcare premises or assets to accommodate the changing requirements in the future healthcare ecosystems.

IC: I am not an expert in design. I am more coming to 'XR Health' from a practical application perspective. My understanding of the design process has been the built environment knowledge and expertise are inherent in the skills of the coders and designers. Those who built XR solutions have considered the mechanics of the built environment. However, I think the key here is 'considered'. So, I agree with you, having a further concrete input from specialists in terms of architecture, would definitely enhance the experience. A wise observation!

ID	Date (dd.mm.yyyy)	Type	Field	Duration	Notes/Transcripts
D	05.07.2022	VC	Architecture	00:56:13	T

RS: Some people might think that there's not much architects can do for the medical XR because it is literally a virtual environment.

ID: That is a basic assumption. But a lot of people see that the difference between real and virtual environment is gradually decreasing, like the Matrix movie. Metaverse is the new world and things are just beginning to take shape. Everyone has different priorities. Economists talk about cryptocurrency and NFTs. Architects can have more freedom to design, invest, and develop some cool experiences for people as we understand what the human-centred design is. This is the way I perceive the virtual environment; we are shaping the experiences in it. Mental health services will be a good starting point as they require fewer physical settings compared to other clinical practices.

RS: You wrote a thesis on how telemedicine will impact the healthcare facilities.

ID: I took the same approach as you do now. My proposition was that there are some physical requirements for telemedicine such as lighting, temperature, and equipment. So, I made several suggestions on how to optimise the healthcare facilities to ensure the best use of telemedicine. At the last part of dissertation, I mentioned that everything will happen remotely although large medical devices will be needed for the treatment of some diseases.

RS: Can you explain about your current role in the NHS? Do you see anything happens within NHS relating to the medical XR and metaverse? Do you use a digital twin?

ID: I work as a CAD manager in the asset management team. Most of our day-to-day tasks is keeping the digital version of building information up to date. I do not think anything will happen in a near future. Now we just use the most basic features of digital twin for digitising the NHS properties.

RS: What is your vision of future healthcare ecosystems?

ID: We can learn from how e-commerce changed the industry. We can look at what Amazon and Alibaba did. They keep most of their logistics facilities. The healthcare sector will use Urban Air Mobility (UAM) to reduce the cost and time associated with medical logistics. Small and easy tasks can be delivered virtual. But large hospitals will always need to keep high-tech and heavy medical devices. As more digital and logistics technologies advance, there will be more user experience-focused programmes in the future hospitals just like many e-commerce businesses now focus on their clients' experiences.

RS: Any other aspects you want to talk about?

ID: Personally, I am sceptical about the idea of combining cryptocurrency with medical metaverse as we now see so many problems of cryptocurrency. But those who have invested in cryptocurrency can benefit from the mental health services in medical metaverse. It's a very stressful job.

ID	Date (dd.mm.yyyy)	Type	Field	Duration	Notes/Transcripts
E	11.07.2022	VC	Computer Science	00:43:50	T

RS: Can you explain about your experience with XR, HCI and sensor/sensing technology?

IE: One of the examples I can give you is that we've put IoT sensors that everybody's doing in the house. We monitor things like movement, humidity, and temperature. For example, we were looking at people living with dementia. One of the things about dementia that you can function well, but you sometimes have problems with your memory and thought process. So,

one of the things that their family want to know if they maintain their hygiene. For example, do they have a shower? And we were able to detect is by having a humidity sensor. In the bathroom, we could see that between 9:00 and 9:30 every day almost to the minute, the humidity in the bathroom would go up, which implies the shower was coming down. We don't know if the person is switched the water on and just left it, but they wouldn't just go in for 15 minutes. So, we could detect that they were having a shower during this time. Also, we detect irregular patterns with the movement sensors. You're 80. You go into your room to sleep at 10:00 at night. If you go into the bathroom and stay for 3-4 hours. This means you've eaten something bad or maybe you fallen because it's not natural to stay in the bathroom for three or four hours. We've been able to detect if the kettle is over boiled or to see everything is normal. We monitored if people are getting enough natural sunlight by putting the light sensor in their rooms. The blinds were not closed all the time because we don't want people to suffer from depression. And obviously this is going into the subject of built environment which wasn't the reason the device was made for. And it made the device look ugly because you need to put batteries in. That's the kind of issues we were coming up with. It's very difficult to make them look nice and be functional.

RS: Other than sensing technology?

IE: We've just started a project dealing with robots for home. We did a few tests with a humanoid robot called 'Pepper'. It's very expensive and didn't really pass a lot of tests we performed. But a lot of people are looking at the robots for home. Our studies have shown that any kind of thing in the house requires attention like petting and feeding from someone makes the older people feel more useful. For example, if old people interact with animals in their homes, then they would walk more and feel more sense of purpose, so their health would get better. So, we are trying to make a 'needy robot'. For example, the robot speaks to you, "Clara, my sensors say that it should be nice and sunny outside. But I can't see outside. So please can you check outside the window and tell me if it's nice and sunny?". So, the person would look out the window and say, "No, it's raining". The, the robot would say, "Oh dear, I got that wrong. Thank you for letting me know. Maybe, I'll switch on the television to find something a bit more reliable. Or can you tell me a story, perhaps from when you were younger? What was it like growing up?". And the robot would record it. These stories will become clues to find out keywords as machine learning.

RS: Can we have the robot now?

IE: We haven't gone to that stage yet. But I've got a PhD student who just started working on this project. We can apply this to a built environment. Have you seen the Roomba, a vacuum cleaning robot? But it's got some sort of problems when they do the generic everyday cleaning. For example, my House has a lot of steps and stairs. And for the foreseeable future these robots will not be able to transfer from one room to another if there is a step. There is Boston Dynamics, Tesla, and all other robots coming, but they will cost about £25,000 within 10 years. There are four by the time they become affordable, it's not going to be 20-30 years when it becomes affordable to anybody that wants to buy that vacuum cleaner. It is very likely that the penetration of robots that have the dexterity to move around and do whatever you want is not feasible. Most of the robots we want are not there to clean your dishes. They do more things for you, sort of mental capacity. So, one thing that we want to do with this needy robot is to understand the people's health.

RS: How?

IE: The robot eyes can detect if people's mobility is fine as they can walk. Then the robot asks people to look out the window and say what they think now. People here do two things, not only walk through and feel healthy, but also look outside the window and understand what the robot says, which means they interact with it. This means we can also detect their mental capacity, while doing the transaction people like. Are they OK? Did they respond OK on time? So, we know immediately that that person is mentally and cognitively fine. We can then push this because there is research that shows you can also detect dementia symptoms. For example, so if you take recordings periodically during time for several years, you can start detecting some changes that might point to some conditions such as dementia.

RS: So, your research deals with dementia care and support as well. When I went through your publications, I came across a series of research helping people with visual impairment.

IE: Yes, I do. A lot of this. The ability research is one of the things that we've done such as visual impairments, people with muscular degeneration, and central vision loss. We researched how VR can help people to do better. A lot of our participants were older people because those conditions hit.

RS: We have talked about sensing and robot technologies. Any relevance to the built environment?

IE: Yes. Will the built environment have a big thing with the robot thing? Because it is the step one problem we're having with our new robot. The type of surfaces and something related to the edge detection to stop the robot from crashing into it can be the issues.

RS: Does the robot collect data to predict the conditions of surrounding environment?

IE: It is not to just predict that, but also the other way around. We can think of an environment which lets the robot and sensors function seamlessly and doesn't get in its way.

RS: Can we use the data sets the robot collected to design the space? I mean, the data sets indicate what the best conditions of built environment to aid people with disability or disorder. Does this make sense?

IE: It does. In one or two weeks, we are going to a place called Gloucester City Homes, a housing association. The government came to them and said that they are thinking of refurbishing buildings to provide older people with healthy life and asked for some guidance as to what technical requirements should be integrated into the building design to futureproof them. And the housing association turned around and said that they don't know for at least 20-30 years. Knowing what technologies are needed for the house is a big thing. I think that's where the key is for. The answer is we don't know. We have all these studies being done in the residential buildings for healthy ageing and people with dementia. But I don't think anybody has taken this. From the gaps we've seen, we can do for example, it looks like we're going to need wider doors, we need a lift if people go walk into this way. This is the kind of stuff that we can. But most things that are already built and inaccessible, they will stay inaccessible. There's a lot of projects because they would do it. That's the way it was done 50-100 years ago.

RS: What about the future environment?

IE: What is the future of the built environment when it comes to immersive technologies? Can they access these places virtually? We can give them access to VR 360° tours of places. Then, can you get a virtual presence? What about a drone or a robot? Navigating! I am from Cyprus and there's a lot of ancient things that you can go and visit. If people are in a wheelchair, it

would be impossible to get to these places. Someone can operate a drone to give people a virtual journey. There are all sorts of things that we can do.

RS: Can you explain more about the VR for people with visual impairment?

IE: The VR research you've seen came from people with trouble reading or watching TV. He couldn't see unless he gets very close to the TV because of the central vision loss. So, he needed to go on the side and look on his peripheral vision. If he watches TV with his family, he has to sit very close to the TV, while his family sit on the couch and he's in their way. He needed a VR device with a video player, something we made for him. So, he can sit on the couch with his family, watching the TV and holding his wife's hand. There are all these things that we can facilitate. We are now working on a model that enables people to sit on their couch, pick up a book from their own library, then the device scans the text and presents it back to them. But people can get the feeling of the book.

RS: So, it is about removing physical constraints.

IE: Yes. I am throwing a lot at you now. I am not sure if you will benefit from all this. So, I am just throwing whatever I know and what I am doing.

RS: What about the standardisation?

IE: XR is struggling because there's no standardised way of doing it. People with dementia can wear glasses to remind them what to do and where things are. There are lots of things we need to think about, and what the built environment should be, specifically for people with disabilities. It's heightened with people's healthy ageing because they will fall under a lot of these disabilities, including auditory, visual motor, cognitive disabilities. We will have more older people with all these spectrums. Anything else I can think of is less impactful and quite weak. So, if you look at one thing and how to change the environment with one thing, there is no guarantee that one specific model is going to be implemented in 3-5 years. It might die.

RS: How do you deal with health regulations to prove the device is safe for the patients?

IE: Critical decisions on medical safety create huge barriers in moving technology forward. The stuff that we do does not directly link to predicting health from a medical perspective. Otherwise, you need to get a lot of licenses. The people dealing with emergency logs don't talk

to a doctor and say I'm going to send you the data. You make a prediction as to if this person has dementia or if the reduced mobility means that they must get an appointment with you. From the moment you start making medical judgments. In terms of giving data that will inform a medical decision. You get into a lot of trouble. Not a bad thing, but it just it. It's very difficult to get into that area because it's safety critical and if your data is for any reason a little skewed or isn't accurate, you are liable. Now, we are not looking at medical decision-making apparatus and emerging technologies. We are looking at technologies that inform family, friends, even medical practitioners, but we are not claiming that they give any sort of medical directive to any medical professional. I am working on a project, but we are giving medical professionals data for them to make a judgment and it required years. Of ethical approval, years of lots of scrutiny. So that's a very dangerous and difficult territory to get into. It is much more likely that emerging technology will be assistive. My advice to you is to be very careful of making any medical decisions based on it. Use it as an assistive technology. Then you are not liable. So, being assistive is better and most of the technology coming out in the future will be assistive rather than medical. It is there to give some information back, rather than to act in a way. So, if the robot detects someone falls down, then we can shout out or the robot can ask if people is okay. Instead of calling ambulance, the robot can call their family or friends. Regarding the robot’s malfunction, we need to avoid using specific materials. There will be a lot of technologies to detect things with heat, radio, or sonar. So, all these things need to be taken into account.

RS: So, we need to make the built environment works for the use of technologies to help people. Can you tell how XR will affect the future healthcare systems? (Researcher explained about the dissertation topic and research questions)

IE: I don’t know much about that. The only thing that I’ve done with healthcare facilities is regarding dentist visits for children. They fear of a new space because they are unaware of the built environment. So, we virtually modelled the space. We took a 360° camera and virtually took pictures of all the journey from entering the door, going up the stairs, and going into the consultancy rooms where the procedure will be undertaking. Then we gave children a virtual tour to see the areas and we put virtual characters who can speak to children so that children can get familiar with the environment. It reduced distress a little bit. At least, children have one less thing to worry about. That’s the only thing that I’ve been involved in terms of an actual healthcare setting. I don’t know if that’s very useful.

RS: It is very useful. You added features to the virtual environment to improve the clinical outcomes by reducing patient's stress.

IE: Exactly. And we envision XR will become a powerful tool because everybody is dealing with it and trying to come up with all different things. For example, sometimes you don't know where the exit is. If you went 15 years ago to the Louvre in Paris, trying to find out how to get out was a big problem. Augmented reality can be useful in this. But you need a good connection. You need to be able to look around and download a huge data quickly. I am guessing one of the things that will be prevalent in the future is that minimalist will be more of thing. The more you overcrowd, the less ability you give any technology. Anything moving around and you're looking around, you can crash into things that are less space free to move around. This comes from sci-fi, but if you look the Ex Machina movie, there's sort of truth in what they build. Robotics and everything require kind of clean, smooth, and quite minimalistic environment. You need a lot more minimalistic environment in a big space. Also, we can think about the ceiling, which is very underutilised space in any buildings. If we need to put anything, it can go up. Can we create some sort of arts in the ceiling to entertain or inform people? We need to think how we interact with it. Try to find a gap, think about requirements, then put them into one place so that we can see what the commonalities are and figure out how we futureproof them.

ID	Date (dd.mm.yyyy)	Type	Field	Duration	Notes/Transcripts
F	19.07.2022	VC	Architecture	00:52:13	N

RS: Built environment experts always work with various stakeholders to design and build the healthcare facilities. We understand the dynamics of stakeholders and what the priorities are in clinical settings. Can we say that we architects understand what is really needed for the healthcare ecosystems in the clinical environment?

IF: No. Architects are only very small part of complex and extensive healthcare systems. Moreover, stakeholder dynamics and project requirements will vary depending on the programmes and stages of healthcare facilities projects.

RS: Can you tell me about your experience with XR?

IF: I have experienced XR not in the clinical settings, but in the design process. We offer 3D interiors walkthrough using VR technology. In many cases, the virtual tour is most effective when conducted with full size mock-ups at 1:1 scale because people are not familiar with either two-dimensional drawings or entirely virtual environment.

RS: Do you use participatory design approach a lot in your work?

IF: You must. Because the participatory design is required to get funding for healthcare facilities in the UK.

RS: Have you seen the Arup's future healthcare ecosystems report? One of my propositions is that we may need to reconfigure the healthcare premises when XR becomes an integral part of the future healthcare systems. Some facilities will become obsolete, we may need a kind of unprecedented facilities instead. As healthcare models evolve, do you see the changes, so to speak a reduction of hospital spaces?

IF: Yes. We may end up with less clinical spaces, more therapeutic spaces. In the hospital, there will be more soft spaces which can be easily relocated in comparatively low cost.

RS: Do you use digital twin in your work?

IF: Not very often, I heard it is very difficult to train and educate people.

ID	Date (dd.mm.yyyy)	Type	Field	Duration	Notes/Transcripts
G	25.07.2022	Email	Healthcare	N/A	N

RS: Does a virtual environment for medical metaverse need to be similarly designed to a clinical space in the real world? For instance, you may think it is necessary to make both clinicians and patients feel familiar and comfortable within the virtual environment or to improve the efficiency of clinical procedures and communications.

IG: It depends on the purpose of configuring virtual environment. If it is for a clinical space, for the kind of we generically think of, a virtual clinic should be designed following the layouts of actual consulting, examination, and treatment rooms because they can be the deciding factors that will affect patients' mental and emotional health.

RS: Does a medical metaverse need to adopt or utilise the flexible and creative nature of virtual environment? For instance, a gamification or an avatar of funny or famous character can be introduced to reduce patients' stress and tension.

IG: The medical metaverse refers to not just a visually experiential space, but also a new spatiotemporal layer in which Clinician-Patient, Clinician-Clinician, and Trainer-Trainee can communicate with each other. The concept of medical metaverse should be more profound yet practical version than ones for game or entertainment. In that sense, the use of avatar or spatial layouts are not critical issues.

RS: Your institution is currently using a medical metaverse for education and research purposes. Another medical college in South Korea has organised the Metaverse Doctors Alliance and invented the own cryptocurrency for their metaverse platforms as inroads into overseas markets. Does your institution have a similar business plan of utilising a medical metaverse? If not, is it because of the practical, technical, or ethical concerns around transmitting people's health-related data through blockchain and NFTs?

IG: Due to the regulatory frameworks or institutional issues of remote care, we are not considering the commercial factors of medical metaverse. I reckon the healthcare sector does not need to become the spearhead to lead such a trend. Rather, we should consider commercialising the medical metaverse after its supporting technologies or markets become finally mature and stabilised. The priority is to develop basic and foundation technologies to create the actual business value of medical metaverse.

RS: On 29 June 2022, the UK government issued a new policy to support the digital health technologies. Considering the potential impacts of digital therapeutics, digital twins, and wearable health devices on the healthcare industry, many scholars, and researchers the future health and care models will become more decentralised, patient-tailored, and preventive. Does the same apply to the healthcare industry in South Korea?

IG: I basically agree that will be a big trend of future healthcare systems. I also think cross-border healthcare services will become eventually possible as the geographical boundaries (between countries) crumble down.

ID	Date (dd.mm.yyyy)	Type	Field	Duration	Notes/Transcripts
H	28.07.2022	VC	Architecture	00:47:55	T

RS: (Researcher explained the topic, research questions, and hypotheses) I took your lecture on the real world and virtual environment. You said it is important to use the same language in terms of spatial layouts for the virtual environment.

IH: First, I can see you discuss your topic by taking interviews. Have you come up with the conclusion yet?

RS: (Researcher explained about the research outcomes and conclusion)

IH: I think your conclusion is too general. Do you focus on the trend or more detailed designing of virtual environment?

RS: I guess that's the limitation of my research. Can I show something? (Researcher showed the Arup's sketches of future healthcare ecosystems) I thought my dissertation was to approach the subject from a big picture level rather than drawing up a design proposal for detailed space layouts.

IH: How about the research findings you gained from those interviews? Now it looks like you just compiled all XR-related information and edited them. Since you study at the Bartlett, I guess you will need to come up with something architecturally meaningful. I don't see them now.

RS: (Researcher shared the findings from previous interviews) I guess I will argue that we need further research on how best to balance the virtual and physical environment. Traditional clinical settings won't work anymore. NHS will have to rearrange their property assets. Something we witnessed from e-commerce or remote work could happen in healthcare industry, I presume.

IH: Perhaps, you can make sort of design proposals in your discussion section. Like a programming stage. Like the others, doctors might need different work settings. And a redesign of patient and staff journeys within the healthcare facility will be necessary. We may need some spaces that we've never seen before because people will start using Urban Air Mobility (UAM) a lot. We cannot prove them now, but we can make kind of frameworks. I think it would be

very interesting if you can draw a picture to visually present your vision in this dissertation because what you are doing is very unique.

RS: (Researcher talked about the difficulty that MXR practitioners currently have and the relevance between the architects' research on HCI and the XR therapy for people with psychosis) At the moment, architects' contribution to this field of MXR is very little.

IH: Based on what I heard from you; I am more convinced that the necessity of cross-sectoral collaboration that involves built environment experts. But eventually, some parts of your dissertation should discuss and visually present how we can apply a new design rule to the virtual space for the future use. It can be one or two pages.

RS: One of your publications studied the roles of illuminance in the virtual environment. Can you explain to me more? Was it to create a sense of presence by reproducing the physical entity in the virtual environment?

IH: No. That was to explain one of many ways of using VR for the design of physical environment. So, it's different from what you are talking about. The simulation of physical environment was to test variables for designing the actual space. However, it applies to the healthcare facilities as doctors need offices, and moreover, lighting is critical element for hospitals in promoting the patient's wellbeing.

RS: (Researcher shared the perspectives of other interviewees from medical, computer science, and business sectors)

IH: I am working with a telecommunication company to create a virtual work platform for them. And one of the issues we have is whether we make the virtual environment look alike the actual space or add some creative elements in it. The real word will keep evolving, and at some point, the space will have very futuristic look. After a long discussion with our clients, we have decided to provide people with a familiar place rather than making it look too crazy or different from its real-life counterpart. Now, how we organise the built environment is still useful to design the virtual spaces. Basically, we had two rules to design the virtual offices. First and foremost, we wanted to make people feel comfortable in the virtual spaces, so we applied the same design principles of actual workplaces to the virtual environment. It will change gradually. I guess the starting point is, like I mentioned in my YouTube lecture on 'the

virtual world and architectural space', that things we feel familiar with spatially will change gradually too. Now, how we organise and create the real-world space is still useful to design the virtual environment. And that's our approach to design the virtual office for the telecommunication company.

RS: (Researcher explained about the possible layouts of future hospitals and changing factors based on the findings from previous interviews)

IH: Even so, we architect can get things ready in advance. We need to be proactive and figure out potentially useful or useless space because we know for sure that technology will enable things in the future.

RS: I understand you are trying to find out the possible roles of architects in the virtual environment. I was curious about your general design approach to it.

IH: Like I said before, architects always wonder how the space will be used. I would take a general approach. We don't need to stick to the law of physics. However, I believe the general standards we've been using for designing the physical environment work well with our spatial cognition because they are proven with certainty through the long and rich history of architecture. We have established those standards for hundreds or even thousands of years. So, I believe they can be a good foundation for designing virtual environment. And we architects are the experts in it.

RS: (Researcher explained about the cybersickness issue)

IH: As technology evolves, we might not need to worry about the cybersickness, and the virtual environment don't have to immigrate the physical environment by the time the technology can solve the problem. The ultimate role of architect is design.

ID	Date (dd.mm.yyyy)	Type	Field	Duration	Notes/Transcripts
H	04.08.2022	Email	Computer Science	N/A	N

RS: Can you explain about your experiences and interpretations of MXR?

II: XR has a wide range of applications towards the medical field. From years of working with disabled or impaired users, typically elderly as a by-product of disability/vulnerability although not my intended research area, I have seen first-hand that this type of technology if utilised properly can bring amazing and dramatic changes to someone's everyday life. My current interpretation when it comes to "MXR" is that the technology is there, is overengineered for what we could use it for, but is heavily underutilised or realised for these kinds of practical implementations. Granted there has been an increasing climb in awareness of XR which feeds straight into MXR, and this was particularly influenced by the covid pandemic where many companies and researchers looked towards remote solutions, which pairs great with XR.

RS: Have you experienced any risks or limitations in your VR research in relation to the built environment?

II: My research has followed some of the earliest VR headsets which may share different risks with the upcoming new VR era (era 3), which focuses on wireless, eye/facial tracking, and additional senses. Since my research has always involved impaired users the testing scenarios have never involved physical movement and instead were seated. One issue with the earliest headsets requiring wires and sensors, however, is not only does the cabling and setup pose a potential health risk (wheelchairs around headset cables, turning, laptops/PCs required to be hooked up to power sources), but the environment itself restricts where VR can be used. Any SteamVR solutions using base stations cannot have reflections in the space, lighting conditions must be accurate, and normally these must be placed in elevated positions for accurate tracking. The same goes for other traditional sensor setups, again requiring extra cabling and space. Restricted environments meant that testing in someone's home was not always feasible, or any business/company/environment that did not have a strong level of neutrality to it. Inside out tracking solutions that some of the more popular headsets use today are a step towards removing this barrier, yet these can still be very sensitive to the environment setup. All these inside out tracking VR headsets today still say they should not be used outdoor or in contact with any sunlight due to risk of camera damage, which is again a limitation.

RS: Did you feel the need of collaborating with built environment experts such as architects, contractors, or property business managers to solve the problems?

II: To a certain level yes. When working with businesses I had to scope out and ensure whatever room was used was suitable, often requiring specific and planned booking and reservation.

RS: From HCI perspectives and in relation to your VR projects, does the notion of 'interactions' between human and computer consider the variables or factors that can be affected by the built environment?

II: In relation to the built environment, as some testing was done comparatively to physical elements, a recreation of the built environment was necessary. This considered variables such as the luminosity levels and distance. To avoid the need of physical movement, distances could be manually manipulated in a controlled scenario that was a replication of the actual built environment.

RS: Can you make suggestions regarding the potential of MXR in healthcare facilities you would think it enables better medical practices or health outcomes?

II: There are almost too many to list. Just within the context of visual impairment, VR devices could be used as highly advanced visual accessibility aids to replace several existing tools and equipment that are relied on currently at a fraction of the price. I have spoken with surgical companies that could use XR as a cost effective and powerful reduction on the reliance of cadaver training. Education, which feeds into health, could vastly benefit in many obvious ways from XR devices. Mental health could benefit greatly from VR providing things like rehabilitation and therapy. One area I am particularly interested in is autism, where VR could not only help us understand someone far better, but also deliver experiences that would otherwise be impossible or very costly; if a visual stimulant is causing sensory overload, we could disable it. Training is an obvious one where almost anything related to medical training could be enhanced through XR devices. Healthcare facilities need to involve tech experts or developers who can suggest and implement solutions to current procedures and practices that would save a lot of time and money, and greater awareness is needed to achieve this.
